# Recent Advances in the Development and Application of Radiolabeled Kinase Inhibitors for PET Imaging

**DOI:** 10.3390/molecules201219816

**Published:** 2015-12-09

**Authors:** Vadim Bernard-Gauthier, Justin J. Bailey, Sheldon Berke, Ralf Schirrmacher

**Affiliations:** Division of Oncological Imaging, Department of Oncology, University of Alberta, 11560 University Ave., Edmonton, AB T6G 1Z2, Canada; jjbailey@ualberta.ca (J.J.B.); sberke@ualberta.ca (S.B.)

**Keywords:** positron emission tomography, tyrosine kinase inhibitors, protein kinases, nuclear imaging, cancer imaging, neuroimaging, fluorine-18, carbon-11

## Abstract

Over the last 20 years, intensive investigation and multiple clinical successes targeting protein kinases, mostly for cancer treatment, have identified small molecule kinase inhibitors as a prominent therapeutic class. In the course of those investigations, radiolabeled kinase inhibitors for positron emission tomography (PET) imaging have been synthesized and evaluated as diagnostic imaging probes for cancer characterization. Given that inhibitor coverage of the kinome is continuously expanding, *in vivo* PET imaging will likely find increasing applications for therapy monitoring and receptor density studies both in- and outside of oncological conditions. Early investigated radiolabeled inhibitors, which are mostly based on clinically approved tyrosine kinase inhibitor (TKI) isotopologues, have now entered clinical trials. Novel radioligands for cancer and PET neuroimaging originating from novel but relevant target kinases are currently being explored in preclinical studies. This article reviews the literature involving radiotracer design, radiochemistry approaches, biological tracer evaluation and nuclear imaging results of radiolabeled kinase inhibitors for PET reported between 2010 and mid-2015. Aspects regarding the usefulness of pursuing selective *vs.* promiscuous inhibitor scaffolds and the inherent challenges associated with intracellular enzyme imaging will be discussed.

## 1. Introduction

Through mediation of crucial signal transduction pathways, the catalytic activity of kinases lies at the cornerstone of a myriad of cellular functions and regulates various cellular processes including differentiation, apoptosis, proliferation and metabolism. The last quarter century has witnessed fundamental discoveries and prominent advances in protein and lipid kinase function and inhibition. The FDA approval of the Bcr-Abl inhibitor imatinib in 2001 and subsequent clinical successes in the treatment of chronic myeloid leukemia (CML) marked the emergence and rapid growth of small molecule kinase inhibitors as a novel therapeutic instrument for cancer treatment [1,2]. As of July 2015, a total of 29 small molecule kinase inhibitors, mostly aimed at tyrosine kinases for the management of neoplastic diseases, have been approved [3,4]. It is remarkable that 20 of those approvals were completed from 2011 onward. Undoubtedly, kinase inhibitors currently constitute one of the most keenly investigated therapeutic classes. Yet, approved kinase inhibitors converge around very narrow pharmacophores and targets, often derived from previously successful inhibitors, and represent only a minute fraction of the structural diversity globally encountered within kinase inhibitors characterized preclinically [1,5]. To date, approved inhibitors have largely left untouched many potential promising applications in areas including the central nervous system (CNS), inflammatory and cardiovascular diseases. Current kinome coverage data from publicly available kinase inhibitors indicate that about half of the 518 human kinases have been targeted, with inhibitors well distributed among the seven main protein kinase groups (AGC, CAMK, CK1, CMGC, STE, TK and TKL) [6,7]. Despite this great progress, the human kinome remains mostly unmapped terrain, providing ample emerging possibilities for drug development in the field of kinase inhibitors [8].

**Figure 1:**
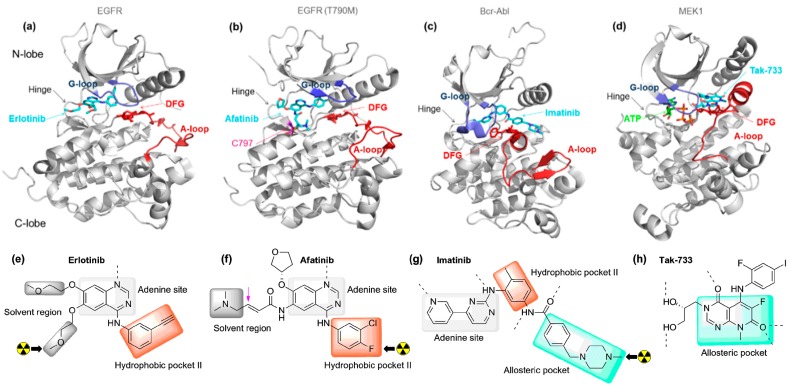
Representative examples of the primary types of small molecule kinase inhibitors including a DFG-in irreversible inhibitor. Kinases are shown in grey, the activation loops in burgundy, the glycine-rich loops in blue and the DFG motif in red (sticks). The carbon atoms of the inhibitors and the ATP are respectively represented in cyan and green (**a**–**c**); The chemical structures and schematic binding modes of the inhibitors are depicted. For inhibitors that have been radiolabeled for PET imaging, the position of the radionuclide (carbon-11 and fluorine-18) is indicated with a black arrow (**e**–**g**); (**a**,**e**) Type I inhibitor; co-crystal structure of erlotinib bound to epidermal growth factor receptor (EGFR) (DFG-in) (PDB ID: 1M17); (**b**,**f**) Type I inhibitor (irreversible); co-crystal structure of Afatinib bound to EGFR T790M (DFG-in) (PDB ID: 4G5P); (**c**,**g**) Type II inhibitor; co-crystal structure of imatinib bound to Bcr-Abl (DFG-out) (PDB ID: 1IEP); (**d**,**h**) Type III inhibitor; co-crystal structure of Tak-733 bound to MEK1 (DFG-in) (PDB ID: 3PP1).

Regardless of sequence differences, protein kinases share common tertiary structure components which include a highly conserved ATP-binding site located at the interface of N- and C-lobes. This binding cleft constitutes the primary anchorage site of most inhibitors (Figure 1a–d) [9,10]. Within the hinge region, a “gatekeeper” residue regulates access to an adjacent hydrophobic pocket. Additional structural features include a glycine-rich loop and a flexible activation loop within the C-lobe initiated by a conserved Asp-Phe-Gly (DFG) motif which dictates the accessibility of the catalytic site. Inhibitors either interact reversibly or irreversibly with kinases. Irreversible inhibitors which bear a Michael acceptor fragment covalently react with a cysteine residue in the environment of the ATP-binding site (Figure 1b) [11,12]. Otherwise, most inhibitors exert reversible binding and are organized in distinct groups according to their binding mode and DFG orientation. Type-I inhibitors engage in ATP-competitive interaction typically binding at the hinge region, with the Asp residue from the DFG motif directed towards the ATP binding site (Figure 1a; DFG-in inhibitors). Type-II inhibitors bind the inactive kinase conformation in which the DFG motif is rotated and allows interaction with an additional allosteric pocket while maintaining contact with the hinge residues (Figure 1c; DFG-out inhibitors).

Type III inhibitors interact with the allosteric pocket without overlapping with the hinge region (Figure 1d). In addition, more rarely encountered type IV inhibitors exploit binding sites distal from the ATP site [13,14].

Despite the relatively high response rates encountered within this drug class, only a minority of patients will ultimately benefit from kinase targeted therapy, due to target expression/engagement, mutation status and pharmacokinetic parameters [15]. Moreover, the initial response rates are hampered by drug resistance occurring over the course of treatment [16]. The identification of treatment-responsive patient subsets for a given inhibitor, and the validation of the most suitable lead candidates from emerging generations of inhibitors early in clinical settings constitute key challenges associated with the advancement and clinical use of kinase inhibitors. In this context, the implementation of nuclear imaging protocols as part of the clinical assessment of treatment response and the drug development process has emerged as a promising approach [17,18,19]. Positron emission tomography (PET) imaging is among the most successful *in vivo* imaging technologies currently in use. PET imaging relies on the coincidental detection of γ rays ensuing from annihilation events triggered by positron emitters incorporated into biologically relevant molecules. Small molecule radiotracers for PET are typically labeled with ^18^F (*t*_1/2_ = 109.8 min; 97% β^+^; E_max_ (β^+^) = 0.64 MeV) or ^11^C (*t*_1/2_ = 20.3 min; 99.8% β^+^; E_max_ (β^+^) = 0.96 MeV) and generate high resolution three-dimensional images showing the dynamic distribution of the molecular probe, thus allowing for the localization and quantification of specific biological targets non-invasively. PET imaging has found broad applications in oncology with the use of ^18^F-labeled 2-deoxyglucose ([^18^F]FDG) for the assessment of tumor metabolic activity. Once injected, [^18^F]FDG is actively transported in metabolically active cell, including tumor cells, and is trapped as [^18^F]FDG-6-phosphate, allowing tissue visualization. In recent years, [^18^F]FDG PET and [^18^F]FDG PET/computed tomography (CT) have been utilized in treatment monitoring imaging for patients undergoing kinase inhibitor therapy [17,20,21,22]. This approach though provides an indirect assessment for kinase inhibitor treatment response. A more targeted approach relies on the PET evaluation of radiolabeled small molecule inhibitors. Radiolabeled isotopologues of approved kinase inhibitors have the potential to differentiate responders from non-responders undergoing treatment similarly to [^18^F]FDG. However, in this context, and in contrast to [^18^F]FDG, the PET signal of interest ensues directly from the interaction of the radiolabeled inhibitor with one or more kinase targets. In the absence of acquired resistance which may compromise the binding of kinase inhibitors, *in vivo* PET imaging of radiolabeled approved drugs or drug candidates can in theory provide expedient data regarding spatiotemporal protein kinase expression. In addition, data relating to whole body distribution and metabolism of the radiolabeled compound may be obtained [18,19]. Considering the magnitude of the kinase inhibitor field and the number of inhibitor development programs, it is clear that the use of radiolabeled inhibitors in early drug development stages can also assist in target identification and validation, while defining compound prioritization and target engagement based on robust *in vivo* data. From a more fundamental perspective, radiolabeled inhibitors taken from scaffolds under development may offer opportunities in neurology and neuro-oncology to measure brain penetration and to quantify kinase density in brain tissue under normal and pathological conditions.

As for radiotracers in general, stringent criteria have to be met in the design of radiolabeled protein kinase inhibitors [23,24,25]. These requirements are valid for the translation of both approved inhibitors and compounds selected from preclinical screenings. First, the intended molecule should display high affinity (typically low nanomolar to picomolar) for its target(s). This is of particular importance for ATP-competitive kinase inhibitors targeting kinases with low K_M, ATP_ values (*vide infra*) due to the high cellular ATP concentrations (1–5 mM) [26]. Also, it is essential that the compounds be efficiently radiolabeled in high specific activity (SA) and useful radiochemical yields (RCYs) following a time-efficient procedure suitably matching the short half-life of the radionuclide used (^18^F, ^11^C, or ^64^Cu). Kinase inhibitors are advantageous in this regard as they often contain readily available positions for labeling with ^11^C and ^18^F. For example, of the 29 approved inhibitors to date, 15 bear at least one readily accessible *O*-CH_3_ or *N*-CH_3_ fragment for radiolabeling through conventional radiomethylation methods with [^11^C]CH_3_I or [^11^C]CH_3_OTf (see examples in Figure 1e–f). Three of these compounds also contain fluorine atoms in activated *ortho*- or *para*- aryl positions. The majority of the approved inhibitors also bear additional potential, albeit more challenging, labeling sites such as asymmetrical ureas, non-activated fluoroaryl moieties and tolyl groups. Compounds identified in preclinical research can also be selected based on the availability of potential radiolabeling positions. In cases where no labeling position is evident, rational radiotracer design may be straightforward due to available crystallographic and structure-activity relationship (SAR) data—both of which are extensive in this field. It is also possible that a readily accessible radiolabeling position may not be suitable due to metabolic susceptibility. In such cases, ^18^F-defluorination will lead to bone uptake while heteroatom demethylation, which is one of the primary CYP450 metabolic pathways, may liberate reactive ^11^C-labeled side products, reducing signal-to-noise ratios and confounding the target-specific PET signal. It is therefore important to include the choice of position for labeling in a larger perspective which includes such *in vivo* considerations. Another important element to consider is selectivity. A large fraction of approved kinase inhibitors are not selective compounds [27,28]. While multitargeted inhibitors may still translate into useful PET radiotracers in terms of biodistribution, metabolism and tumor imaging studies, their application outside of a “drug validation” paradigm may be more limited. Finally, when considering brain imaging with kinase inhibitors, multiple physicochemical properties including molecular weight, lipophilicity, polar surface area and hydrogen bond donors become increasingly important due to the restrictive blood brain barrier (BBB) [29,30]. In previous years, excellent reviews have covered the topics of radiolabeled kinase inhibitors for PET imaging [25,31]. Within such a rapidly growing field, the present review summarizes the work accomplished within the last 5 years and provides an overview of the current stage of the field.

## 2. Synthesis and Evaluation of Radiolabeled Small Molecule Kinase Inhibitors for PET Imaging

### 2.1. Radiolabeled Tyrosine Kinase Inhibitors

The ErbB tyrosine kinase family is composed of analogous receptors which include EGFR (Erb1B), HER2/neu (human epidermal growth factor receptor 2, ErbB2), HER3 (human epidermal growth factor receptor 3, ErbB3) and HER4 (human epidermal growth factor receptor 4, ErbB4). It is arguably the most investigated kinase group both in terms of signaling pathway mapping and drug development. Multiple inhibitors targeting ErbB receptors, which are often overexpressed or mutated in human cancer, have progressed into clinical research. Such compounds tend to bear a 4-anilinoquinazoline core as a preferred hinge-binding motif. Expectedly, 4-anilinoquinazoline-bearing ErbB inhibitors were also the first and most explored radiolabeled inhibitor class for PET imaging applications [32,33,34,35,36,37,38,39,40,41,42,43,44,45,46,47,48,49,50,51,52]. Current applications in this class have converged around ^11^C- and ^18^F-isotopologues of clinically approved inhibitors which in some cases has helped fast-tracking the translation towards human imaging applications.

Gefitinib (Iressa^®^, AstraZeneca) is a selective single-digit nanomolar EGFR inhibitor approved as a third line of treatment in patients with non-small cell lung cancer (NSCLC). Positive clinical response to gefitinib treatment is primarily dependent on two EGFR-activating mutations leading to ligand-independent activation (the L858R mutation and exon 19 deletions). These mutations significantly reduce ATP affinity for EGFR while simultaneously increasing inhibitor affinity which elicits a favorable initial treatment response [53].

**Figure 2:**
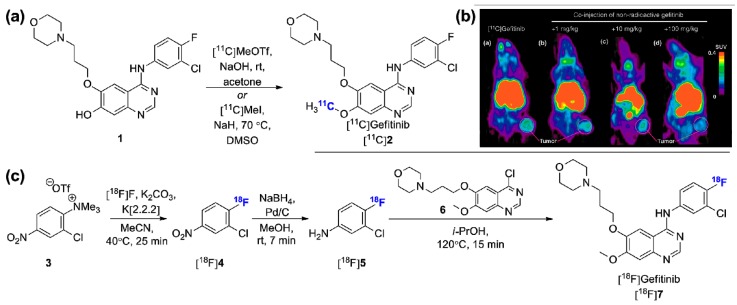
Radiolabeled gefitinib. (**a**) Alternative conditions for the radiosynthesis of [^11^C]gefitinib; (**b**) *In vivo* PET evaluation (coronal images) of [^11^C]gefitinib in tumor-bearing (NFSa) mice at baseline (**a**) and with increasing non-radioactive gefitinib (**b**–**d**) (adapted with kind permission from Springer Science + Business Media: [59], Springer International Publishing^©^ AG); (**c**) Radiosynthesis of [^18^F]gefitinib. (SUV: standardized uptake value).

Subsequent mutation events (e.g., T790M) partially restore the affinity of ATP for EGFR and impair type-I EGFR inhibitor treatment such as gefitinib or erlotinib (Tarceva^®^, OSI Pharmaceuticals), inevitably leading to resistance. Of importance, only 5%–20% of NSCLC patients carry EGFR-activating mutations [54]. Therefore, the pharmacokinetic evaluation of gefitinib and tumoral EGFR status imaging has been the initial driver in the development of radiolabeled versions of gefitinib [55,56,57,58,59]. [^11^C]gefitinib ([^11^C]**2**, Figure 2a), obtained via [^11^C]CH_3_I or [^11^C]CH_3_OTf methylation of precursor **1** was evaluated in fibrosarcoma (NFSa)-bearing mice [59]. The murine origin of the NFSa cell line limits the relevance of the study in the context of human cancer. Moreover, although the tracer was shown to be stable and accumulated specifically in the tumor model (Figure 2b), *in vitro* characterization of the NFSa cells failed to detect EGFR which suggests that *in vivo* specific tumor binding in this case may not be related to EGFR expression. Those results are in line with previous reports involving [^18^F]gefitinib. In this case [^18^F]gefitinib can be obtained following a 3-step procedure starting from the trimethylammonium triflate precursor **3** (Figure 2c). An automated reliable procedure for routine production was recently reported which provides [^18^F]gefitinib in 17.2% ± 3.3% RCY (decay corrected, d.c., *n* = 22) and >99% radiochemical purity following a 2.5 h procedure [60]. Despite the lengthiness of this approach compared to the ^11^C-labeling, the longer half-life of ^18^F was justifiably considered advantageous. Unfortunately, *in vivo*, despite suitable *in vitro* profile and *in vivo* stability, [^18^F]gefitinib failed to display uptake in various xenograft models derived from EGFR-expressing human cell lines including treatment responsive models (H3255 and H1975 cell lines) [56]. Both high non-specific binding due to the lipophilicity of the radiotracer, and efflux susceptibility from the ATP-binding cassette (ABC) transporter ABCB1 (P-glycoprotein) and ABCG2 (breast cancer resistance protein) have been put forward to explain the observed results [25,56]. These transporters are highly expressed at the BBB, in excretory organs, and in many tumors and they constitute the primary efflux proteins responsible for the reduction of intracellular xenobiotic levels. Expression of ABCG2 is a well-known drug resistance mechanism in gefitinib treatment [61]. In light of the positive results obtained with [^11^C]erlotinib which has similar lipophilicity, discussed below, it appears likely that the lack of EGFR-specific signal in microdosing PET experiments with [^11^C]/[^18^F]gefitinib ensues mainly from efflux mechanisms. In fact, whereas both gefitinib and erlotinib are known dual ABCB1/ABCG2 substrates, gefitinib efflux susceptibility is significantly more pronounced compared to erlotinib, as demonstrated by comparative MDCKII permeability experiments [62].

**Figure 3:**
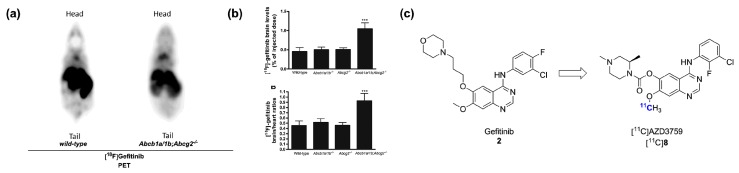
From gefitinib to AZD3759. (**a**) Representative PET images of [^18^F]gefitinib in wild-type and *Abcb1a/1b*;*Abcg2^−/−^* mice; (**b**) Comparative quantitative results of brain uptake in wild-type, *Abcb1a/1b*^−/−^, *Abcg2*^−/−^ and *Abcb1a/1b*;*Abcg2*^−/−^ mice (*** *p* < 0.0001, summed 30–60 min post i.v. [^18^F]gefitinib injection) (Images in (**a**,**b**) adapted from [64] with permission from Elsevier); (**c**) Chemical structure of [^11^C]AZD3759.

Taking advantage of these observations, two distinct studies, first using [^11^C]gefitinib [63] and more recently using [^18^F]gefitinib [64], have explored the ABCB1/ABCG2-mediated brain penetration of radiolabeled gefitinib. In the most recent study, the brain penetration of [^18^F]gefitinib was shown to be limited by both transporters in a synergistic manner using *Abcb1a/1b*^−/−^, *Abcg2*^−/−^ and *Abcb1a/1b*;*Abcg2*^−/−^ mice (Figure 3a,b). Pretreatment with the dual ABCB1/ABCG2 inhibitor elacridar (10 mg/kg) led to enhanced brain penetration of the radiotracer in wild-type mice. With the limited availability of ^18^F-labeled PET radioligands targeting both ABCB1/ABCG2 and the promising clinical applications for such probes, this study suggests the repurposing of [^18^F]gefitinib as an imaging agent for the assessment of ABCB1/ABCG2 activity in the context of CNS diseases. [^18^F]Gefitinib may also be applicable for the quantification of drug-drug interactions (DDIs) at the BBB. As it appears likely that upcoming studies will explore these avenues, work towards the validation of this potential application will be positively influenced by the fact that gefitinib is already well established in the clinic.

Most recently, AstraZeneca reported on the preclinical development and validation of AZD3759, a brain penetrating orally active EGFR inhibitor currently undergoing a phase I clinical trial in patients with mutation positive NSCLC [62,65]. This lead, derived from gefitinib, was rationally developed in order to mitigate the efflux transport at the BBB and offer a new line of treatment for the significant portion of NSCLC patients who will ultimately develop CNS metastases. A radiolabeled version of this inhibitor, [^11^C]AZD3759 (Figure 3c), was developed as part of a brain tissue engagement proof-of-principle PET microdosing study in cynomologus monkeys. The radiotracer showed excellent brain penetration in line with the favorable MDCKII permeability results. This preliminary study provides an unambiguous illustration of the potential of radiolabeled kinase inhibitors in drug development. It will be interesting to see if [^11^C]AZD3759 can be applied to the imaging of brain metastases of EGFR inhibitor-responsive NSCLC cases or if it will be synthesized as an ^18^F-labeled tracer in the near future. The identification of [^11^C]AZD3759 also provides an alternative to ABCB1/ABCG2-substrate radiolabeled EGFR inhibitors for peripheral tumor imaging and could be used to validate the hypothesis that ABCB1/ABCG2 interaction has been the key determinant in the failure of [^11^C]gefitinib and [^18^F]gefitinib for peripheral tumor imaging in preclinical models.

**Figure 4:**
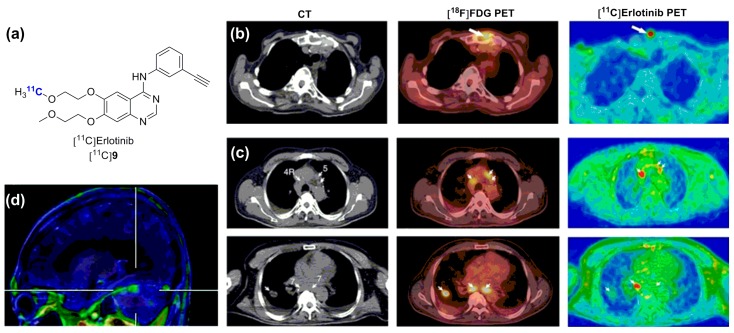
Selected *in vivo* results with [^11^C]erlotinib. (**a**) Chemical structure of [^11^C]erlotinib and comparative PET imaging studies; (**b**) Bone metastasis (**c**) and tumor/lymph node metastasis accumulation of [^11^C]erlotinib in non-small-cell lung carcinoma (with CT and [^18^F]FDG PET) (Figures in (**b**,**c**) adapted by permission from Macmillan Publishers Ltd on behalf of Cancer Research^©^, UK, [70] 2011); (**d**) Magnetic resonance imaging-PET image from [^11^C]erlotinib accumulation in brain metastatic lesions in a patient diagnosed with a non-small-cell lung carcinoma. (CT: computed tomography) (Adapted by permission from [71], from the publisher Wolters Kluwer).

Erlotinib (Tarceva^®^, OSI Pharmaceutical) is a type-I EGFR inhibitor analogous to gefitinib, yet more potent and less selective, which is efficient in similar mutation-positive NSCLC patients (EGFR *K_d_* = 0.67 nM) [27,66]. As mentioned above, erlotinib has been radiolabeled with carbon-11 and evaluated favorably in an erlotinib-sensitive preclinical model using study designs comparable to those used for the radiolabeled gefitinib cases (Figure 4a) [67,68,69]. Rapid clinical translation of [^11^C]erlotinib provided promising preliminary results in two distinct studies with small NSCLC patient cohorts (10 and 13 patients) [70,71]. In a first study, [^11^C]erlotinib was shown to accumulate in malignant lesions and lymph node metastases of undefined mutation status. Interestingly, variation in [^11^C]erlotinib accumulation in the primary tumor and metastatic sites within the same subject was observed, suggesting that a single biopsy may provide incomplete data for EGFR profiling, supporting the development of a [^11^C]erlotinib-based imaging procedure for EGFR status assessment (Figure 4b,c) [70]. In a following proof-of-concept study, the correlative relationship between EGFR mutation status, in this case the 19 exon deletions, and [^11^C]erlotinib tumor uptake was demonstrated [71]. Interestingly, [^11^C]erlotinib was also shown to accumulate in an erlotinib-responsive brain metastatic lesion from a patient harboring an erlotinib-sensitized exon 19 deletion within the primary lung tumor (Figure 4d) [72]. The potential of [^11^C]erlotinib to image brain metastases in this context may be driven by the fact that those patients may already present a compromised BBB. Collectively, these three studies supported further investigation towards PET-driven personalized therapy based on the EGFR status in larger clinical studies. However, Traxl *et al.*, recently presented data evidencing potential problems with the use of [^11^C]erlotinib for this purpose [73]. [^11^C]Erlotinib brain distribution was shown to be substantially superior in *Abcb1a/1b*;*Abcg2*^−/−^ mice than previously described with [^18^F]gefitinib. Elacridar pretreatment (10 mg/kg) in wild type mice restored brain uptake levels comparable to *Abcb1a/1b*;*Abcg2*^−/−^ mice, an effect which was only partial using the same dosing with [^18^F]gefitinib. This difference may be related in part to the fact the [^18^F]gefitinib study [64] used a therapeutically relevant [^18^F]gefitinib dose (1 mg/kg) whereas the [^11^C]erlotinib/elacridar data was derived from [^11^C]erlotinib microdosing experiments. An important observation from the work by Traxl and colleagues, which may have important clinical significance for the use of [^11^C]erlotinib, is the fact that the distribution of [^11^C]erlotinib in peripheral, and probably tumor tissues, was affected by ABCB1/ABCG2. It follows that erlotinib displays non-linear pharmacokinetics due to efflux transporters and that it may be challenging to derive information regarding erlotinib distribution at therapeutic doses from [^11^C]erlotinib disposition in PET studies. This should be a factor to address in upcoming broader validations of [^11^C]erlotinib in NSCLC patients.

**Figure 5:**
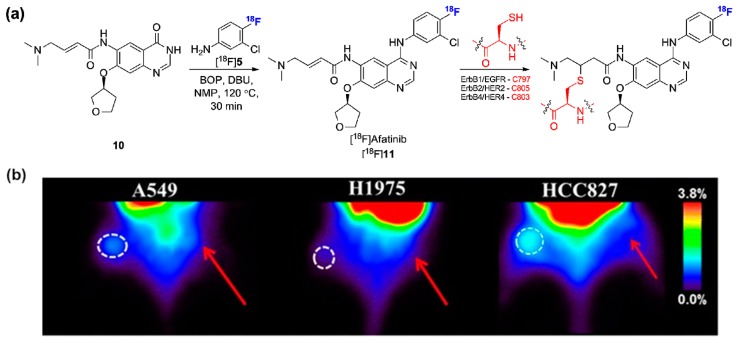
(**a**) Radiosynthesis of [^18^F]afatinib and chemical structures of the irreversible covalent adducts upon interaction at the ATP binding sites of the ErBb protein family; (**b**) Comparative coronal PET images of [^18^F]afatinib in lung cancer-bearing mice with different EGFR mutation status. Red arrows indicate reference tissue. (This research was originally published in *EJNMMI Research*, [74]).

The comparative *in vivo* evaluation of [^11^C]erlotinib with the irreversible inhibitor [^18^F]afatinib has been recently described in mutation sensitized and wild-type EGFR-expressing tumor bearing mice [74]. Although irreversible radiolabeled EGFR inhibitors have been previously described, *in vivo* data and preclinical validation in relevant tumor-bearing models have been limited so far [43,44,45,46,47,48,49,50,51]. Afatinib (Gilotrif^®^, Boehringer Ingelheim) is a type I EGFR, ErbB2/HER2, and ErbB4/HER4 inhibitor, which displays low nanomolar affinity for EGFR^L858R/T790M^ in contrast to first generation EGFR inhibitors. Afatinib covalently and irreversibly reacts with hinge proximal cysteine residues (Figure 5a) [75]. Afatinib also displays off-ErbB selectivity comparable to first generation inhibitors such as gefitinib [75]. The radiosynthesis of [^18^F]afatinib employed a strategy reminiscent of the approach devised to obtain [^18^F]gefitinib (Figure 5a) with the difference that the 3-chloro-4-[^18^F]fluoroaniline ([^18^F]**5**, Figure 5a) intermediate was used in a BOP-mediated condensation reaction with 4-quinazolinone precursor 10 instead of a chloro precursor due to the presence of the Michael acceptor side chain [76]. Despite the rather long radiosynthesis, [^18^F]afatinib was obtained in sufficient RCYs for *in vivo* evaluation (17.0% ± 2.5% RCY d.c.). Initial evaluation in A549 (wild-type EGFR) and HCC827 (exon 19 deletion EGFR) tumors showed moderate tumor uptake in both models (about or under 1%ID/g from 0–120 min p.i.). Unexpectedly, no difference in tumor uptake between these two cell lines was observed. As afatinib is a known P-gp substrate [77], and immunohistochemical staining confirmed P-gp expression in both cell lines, the possible influence of efflux transporters to explain those results were addressed with a comparative evaluation of [^18^F]afatinib with [^11^C]erlotinib under baseline and tariquidar pretreament [74]. Overall, both tracers displayed similar tumor uptake kinetics and tumor-to-background ratios in three tumor models (including A549 and HCC827) at baseline. The only divergence from those results following tariquidar administration was observed in the HCC827 model with [^18^F]afatinib which demonstrated significantly higher absolute tumor uptake (1.9% ± 0.1%ID/g *vs.* 1.2% ± 0.2%ID/g). Concomitant higher background (from contralateral tissue) however led to unaltered tumor-to-background ratios. Selective irreversible inhibitors may be advantageous compared to reversible ATP-competitive inhibitors in terms of residence time [78,79]. It may be of interest to further explore the potential of this binding mode for *in vivo* PET imaging (see Figure 7).

**Figure 6:**
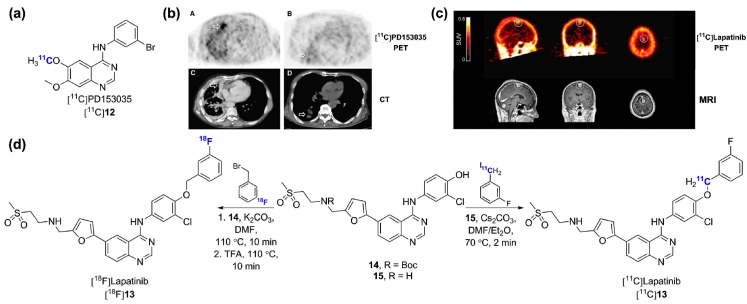
(**a**) Chemical structure of [^11^C]PD153035; (**b**) [^11^C]PD153035 accumulation in a adenocarcinoma (**A**,**C**) and squamous cell carcinoma (**B**,**D**) with corresponding PET and CT images (originally published in [86] © the Society of Nuclear Medicine and Molecular Imaging, Inc.); (**c**) [^11^C]Lapatinib accumulation in a brain metastasis from a Her-2-positive breast cancer patient with corresponding MRI images (originally published in [90] © 2015 Springer); (**d**) Radiosynthesis of [^11^C]lapatinib and [^18^F]lapatinib.

PD153035 is an experimental EGFR inhibitor, prototypical of the 4-anilinoquinazoline scaffold [80]. Along with staurospaurine analogues [81], [^11^C]PD153035 (Figure 6a) is one of the first reported radiolabeled protein kinase inhibitors [34,35,82,83,84]. PET [^11^C]PD153035 has been used in biodistribution and radiation dosimetry studies in humans [85] and PET/CT [^11^C]PD153035 was validated as a noninvasive survival predictor in advanced chemotherapy-refractory NSCLC in a 21 patient cohort undergoing erlotinib treatment (Figure 6b) [86]. A detailed metabolic investigation in rodents was also reported [87]. More recently, preliminary data detailing the potential of [^11^C]PD153035 for EGFR-expressing glioma imaging has been reported [88]. In that study, [^11^C]PD153035 tracer uptake correlated to EGFR expression irrespective of the mutation status. Although this tracer is also likely to be amongst the most investigated radiolabeled kinase inhibitors, more recent research in EGFR imaging has shifted towards approved derivatives. Its further use as a radiotracer for lung cancer or malignant gliomas may also be impeded by the same intrinsic limitations as mentioned above for other 4-anilinoquinazoline-based EGFR radiolabeled inhibitors which were not optimized for efflux transporter liabilities.

Lapatinib (Tykerb^®^, GlaxoSmithKline) is a highly selective dual EGFR, ErbB2/HER2 inhibitor approved in combination therapy for the treatment of HER2-overexpressing advanced metastatic breast cancer. Different to other ErbB inhibitors described so far, lapatinib is a type-II inhibitor which interacts with the inactivated DFG-out kinase conformation of EGFR and HER2 [3]. In recent years, two radiolabeled versions of lapatinib have been described. The radiosynthesis of [^18^F]lapatinib was first reported by Basuli and colleagues and employed a manual multi-step strategy in which the 3-[^18^F]fluorobenzyl bromide intermediate was reacted with the Boc protected intermediate **14** (Figure 6d) [89]. This study did not include a biological evaluation of [^18^F]lapatinib. While the half-life of fluorine-18 is more suitable than carbon-11, a recent clinical study opted to develop a carbon-11 lapatinib isotopologue ([^11^C]lapatinib, Figure 6d) [90]. Since this clinical trial was directed at the exploration of brain and CNS metastasis penetration of lapatinib in breast cancer patients with secondary brain tumors, the carbon-11 option may have been preferred in order to exclude potential confounding factors due to defluorination issues leading to cranial ^18^F^−^ deposition. [^11^C]Lapatinib was synthesized following a two-pot four-step procedure making use of [^11^C]-3-fluorobenzyl iodide (via Grignard reaction with [^11^C]CO_2_) and precursor **15** (Figure 6d). Although the implementation of a Grignard reaction using trace amounts of radiolabeled [^11^C]CO_2_ remains a challenging task, the authors successfully synthesized [^11^C]lapatinib in useful RCYs and satisfactory specific activity following an automated process. The study showed that [^11^C]lapatinib is stable *in vivo* and differentially accumulates in normal brain tissue *vs.* brain metastasis enabling tumor visualization (Figure 6c). With lapatinib being a known ABCB1/ABCG2 substrate [91], it was hypothesized that therapeutic doses could partially saturate these efflux transporters and enhance brain uptake. However, [^11^C]lapatinib PET following lapatinib administration at therapeutic doses did not result in enhanced brain penetration suggesting that prophylactic treatment with lapatinib to prevent brain metastasis formation is likely to prove unsuccessful. It is interesting to note, as did the authors of this study, that the earlier completion of this study would have provided valuable information to a larger concomitant study attempting to determine the possible prophylactic potential of lapatinib in HER2-positive metastatic breast cancer patients [92]. This illustrates furthermore the potential of radiolabeled kinase inhibitors in the drug development process.

**Figure 7:**
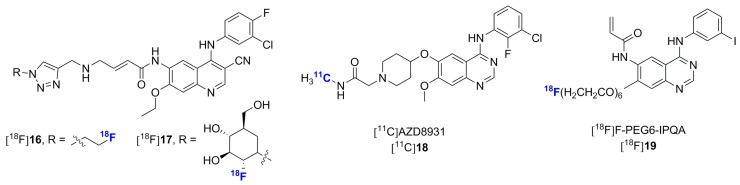
Chemical structures of recently identified and evaluated ErbB-1/ErbB-2/ErbB-3 kinase inhibitors.

Within the last five years, multiple additional experimental type-I ErbB inhibitors exclusively based on the 4-anilinoquinazoline scaffold were synthesized and in some instances evaluated *in vivo* in preclinical settings [49,93,94,95,96,97,98,99,100,101]. In addition to the data presented for [^18^F]afatinib, work towards the development of cysteine reactive irreversible radiotracers have included compounds [^18^F]**16**, [^18^F]**17** and [^18^F]PEG6-IPQA [93,94,95,96] (Figure 7). Of these, [^18^F]**16** and [^18^F]PEG6-IPQA were investigated *in vivo* and were shown to preferentially accumulate in high EGFR-expressing A431 tumor xenografts. Together with the data gleaned from the [^18^F]afatinib preliminary evaluation, these studies failed so far to indicate a distinctive advantage of irreversible over reversible ErbB inhibitors for PET imaging in preclinical models. Novel exploratory ErbB reversible inhibitors include a carbon-11 isotopologue of the clinical candidate AZD8931, [^11^C]AZD8931 ([^18^F]**17**, Figure 7), but no imaging data accompanied the report relating this radiosynthesis [98]. As can be expected, despite in some instances similar pre-clinical data with these experimental inhibitors as compared to approved radiolabeled compounds, these efforts have still remained at preclinical levels years after their original publication. Overall, 4-anilinoquinazoline-based inhibitors remain the only ErbB radiotracer scaffold explored to date. In this context, the radiolabeling and validation of emerging non-4-anilinoquinazoline ErbB inhibitor scaffolds may prove advantageous [102,103,104,105].

Inhibitors of the vascular endothelial growth factor receptors (VEGFR1, VEGFR2, VEGFR3), frequently overexpressed in human cancers, have been extensively explored and proven clinically beneficial for the blocking of critical tumoral angiogenic and growth pathways. Approved inhibitors having VEGFRs as their primary kinase targets include: sorafenib (Nexavar^®^, Bayer), sunitinib (Sutent^®^, Pfizer), pazopanib (Votrient^®^, GlaxoSmithKline), axitinib (Inlyta^®^, Pfizer), regorafenib (Stivarga^®^, Bayer), nintedanib (Ofev^®^, Boehringer Ingelheim), lenvatinib (Lenvima^®^, Eisai Inc.) and vandetanib (Caprelsa^®^, AstraZeneca) [3]. Inhibitors of this class tend to display various levels of promiscuity, disrupting several non-VEGFR kinases in similar nanomolar potencies, including the platelet-derived growth factor receptor (PDFGR), c-Kit, Fms-like tyrosine kinase 3 (Flt-3), RET, TIE-2 and Raf kinases [27,28,106,107,108,109,110]. Despite the fact that the translation of these polypharmacological inhibitors into PET radiotracers may still generate useful results for tumor imaging (where many such kinases may be overexpressed in parallel), these probes are likely to be of limited use as tools to elucidate VEGFR expression selectively. 

**Figure 8:**
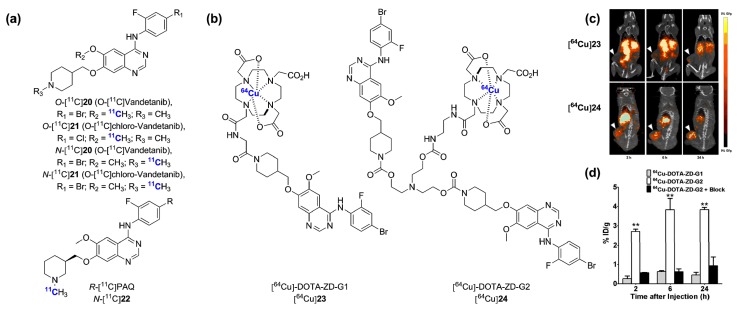
VEGFRs targeted radiotracers. (**a**) Chemical structures of radiolabeled inhibitors primarily targeting VEGFRs: [^11^C]vandetanib, [^11^C]chloro-vandetanib and *R*-[^11^C]PAQ; (**b**) Chemical structures of vandetanib-derivatized ^64^Cu-labeled monomeric ([^64^Cu]**23**) and dimeric ([^64^Cu]24) radiolabeled inhibitors; (**c**) Coronal PET/CT images of inhibitors [^64^Cu]**23** and [^64^Cu]**24** in U-87 tumor bearing mice and (**d**) corresponding quantitative analysis. ** *p* < 0.0001 (this research was originally published in [111] © the Society of Nuclear Medicine and Molecular Imaging, Inc.).

Derivatives or isotopologues of vandetanib [111,112,113], sorafenib [114,115], sunitinib [116] and nintedanib [117] as well as preclinical leads [118,119,120] have been radiolabeled for PET imaging. The initial radiosynthesis and evaluation of the vandetanib analogue *R*-[^11^C]PAQ in the genetically modified metastatic MMTV-PyMT mice model showed only marginal tumor uptake (Figure 8a) [112]. Straightforward [^11^C]CH_3_OTf methylation of suitable des-methyl precursors yielded the *O-*[^11^C]**20**, *N-*[^11^C]**20**, *O-*[^11^C]**21** and *N-*[^11^C]**21** radiotracers, but none were evaluated *in vivo* [113]. A major preclinical contribution by Li and colleagues [111] recently demonstrated the promising potential of a ^64^Cu-labeled vandetanib-based dimer probe ([^64^Cu]**24**) using the U-87MG tumor xenograft model (Figure 8b; *t*_1/2_^64^Cu = 12.7 h) [111]. Although previous EGFR inhibitors have been labeled with ^99m^Tc for SPECT [121] (but not evaluated *in vivo*), this is the first instance of both a ^64^Cu-labeled kinase inhibitor and of the utilization of a multimerization strategy in this context. Receptor binding assays demonstrated a 100-fold affinity improvement for the dimeric probe [^64^Cu]**24** *vs.* a monomeric analogous derivative (44.7 nM for [^64^Cu]**23** *vs.* 0.45 nM for [^64^Cu]**24** in U-87MG cells). Remarkably, a pronounced difference in tumor uptake was observed between [^64^Cu]**23** and [^64^Cu]**24**. While [^64^Cu]**23** displayed very low tumor uptake (0.46% ± 0.06%ID/g, 24 h p.i.), [^64^Cu]**24** showed rapid and lasting specific accumulation into the U-87MG tumors (3.84% ± 0.05%ID/g, 24 h p.i.), (Figure 8c,d). Tumor-to-muscle ratio was optimal at ~5 h post injection (p.i.) (~40) and decreased thereafter, but remained >30 until the last time point imaged (24 h p.i.). Such ratios are significantly superior to what is typically observed with small molecule kinase inhibitors labeled with carbon-11 or fluorine-18 within the allowable scanning time frame permitted by those radioisotopes (typically 60–90 min p.i.). No *in vivo* stability data were provided. With the recent demonstration that free ^64^Cu accumulates in U-87MG tumor tissue *in vivo* (among other tumor cells lines) [122], this data would prove valuable. The success of [^64^Cu]**24** compared to [^64^Cu]**23** was attributed to the favorable synergistic effect of the multivalent inhibitor and the possible prolonged circulation time and slower tumor washout. This unique work raises important questions regarding the value of using longer-lived isotopes to image kinases with small molecule-type constructs. It appears that this type of multivalency platform deserves careful examination in the PET kinase inhibitor field as a whole.

**Figure 9:**
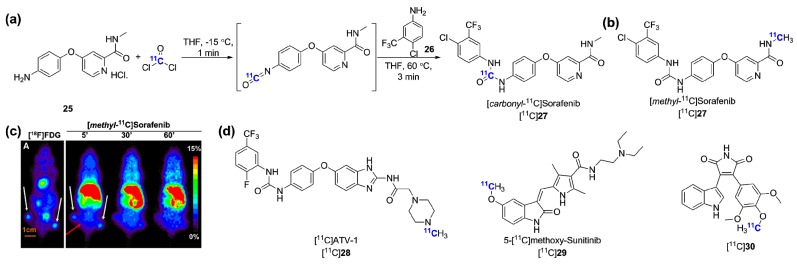
Other radiolabeled multikinase inhibitors targeting VEGFRs. (**a**) Radiosynthesis of [*carbonyl*-^11^C]sorafenib; (**b**) Chemical structure of [*methyl*-^11^C]sorafenib; (**c**) [^18^F]FDG (**A**) and [*methyl*-^11^C]sorafenib coronal PET images of RXF393 tumor bearing mice (reprinted from [115] with permission from Elsevier); (**d**) Chemical structures of additional recently radiosynthesized multikinase inhibitors targeting VEGFRs.

Sorafenib is a multikinase type-II inhibitor which was initially labeled at the carbonyl position using [^11^C]phosgene (obtained from [^11^C]CO_2_ → [^11^C]CH_4_→ [^11^C]CCl_4_→ [^11^C]COCl_2_) by Asakawa *et al.* [114]. The tracer [*carbonyl*-^11^C]sorafenib was obtained in RCYs of 8%–11% (from [^11^C]CO_2_) in a 40 min synthesis using precursor **25** (Figure 8a). No tumor imaging results were presented but injection in *Abcb1a/1b;Abcg2*^−/−^ mice confirmed that sorafenib brain accumulation is limited by both transporters [123]. A second study by Poot and colleagues delineated an alternative route towards [*carbonyl*-^11^C]sorafenib making use a [^11^C]carbon monoxide rhodium-mediated carbonylation reaction [115]. This approach delivered [*carbonyl*-^11^C]sorafenib in higher RCY (27%) than the [^11^C]phosgene synthesis but was abandoned in favor of a more straightforward and reliable [^11^C]CH_3_I methylation synthesis as both radiotracers were shown to be similarly stable *in vivo* ([*methyl*-^11^C]sorafenib, Figure 9b). This alternative tracer, [*methyl*-^11^C]sorafenib, was obtained in 60% RCY (d.c.) and evaluated in three xenografts from cell lines characterized for Raf-1 expression. Of those, only the renal cancer cell line RXF393 xenografts showed modest tumor uptake over the background signal (2.52% ± 0.33%ID/g, 7.5 min p.i.). The limited tumor uptake and tumor-to-background ratios with this probe may be related to its inherent promiscuity and the influence of efflux transporters. Neither data regarding the expression in the tested xenograft models of other high affinity kinase targets of sorafenib (which include VEGFR) nor blocking experiment results were provided. Other radiolabeled multitargeted inhibitors included [^11^C]ATV-1, the sunitinib derivative [^11^C]**29** and the 1*H*-pyrrole-2,5-dione inhibitor [^11^C]**20**. Interestingly, [^11^C]ATV-1 was used in a rat model of myocardial infarction (MI) as a putative preclinical tool to image angiogenesis processes during tissue repair following MI. [^11^C]ATV-1 showed superior uptake in infarct region after MI induction in correlation with Tie-2, PDGFRα and VEGFR-2 expression as validated by immunohistochemistry.

**Figure 10:**
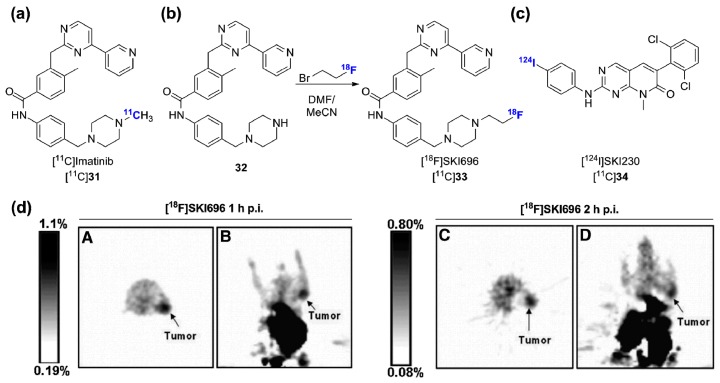
Bcr-Abl targeted radiotracers. (**a**) Chemical structure of [^11^C]imatinib; (**b**) Radiosynthesis of [^18^F]SKI696; (**c**) Chemical structure of [^124^I]SKI230; (**d**) Transverse and coronal [^18^F]SKI696 PET images of K562 tumor bearing mice at 1 h p.i. (**A**,**B**) and 2 h p.i. (**C**,**D**) (this research was originally published in [127] © the Society of Nuclear Medicine and Molecular Imaging, Inc.).

Imatinib was the first FDA approved kinase inhibitor and initially approved for the treatment of CML via Bcr-Abl inhibiton. Imatinib also strongly inhibits c-Kit and PDGFR but shows a fairly good selectivity outside those targets. The DFG-out binding mode of imatinib was a serendipitous discovery that served as a foundation in the development of inhibitors targeting inactive kinase conformations [1]. The initial [^11^C]imatinib PET study delineated biodistribution and pharmacokinetic data including low brain uptake compatible with active transporter efflux in baboons, but was not followed by advanced preclinical experiments using tumor-bearing mice (Figure 10a) [124]. Instead, [^18^F]SKI696, a fluorinated imatinib surrogate [125], and [^124^I]SKI230 [126], were both developed and shown to accumulate moderately in Bcr-Abl overexpressing K562 cell xenografts (human immortalized myelogenous leukemia cells, Figure 10b–d) [127]. [^18^F]SKI696 was synthesized by reacting 1-bromo-2-[^18^F]fluoroethane with precursor 32. Despite detectable tumor uptake (1.2% ± 0.4%ID/g, 60 min p.i.), unfavorable tumor-to-background ratio and high radioactivity uptake in the abdominal cavity were observed (Figure 10d). More recently, preclinical imaging of [^18^F]SKI696 (also identified as [^18^F]STI-575) was revisited by Peng and colleagues [128]. This study confirmed the previous K562 xenografts findings and showed similarly low uptake profile in c-Kit expressing U87WT tumor-bearing mice. In the absence of a clear imaging rationale for Bcr-Abl targeting in CML, and a potentially limited applicability to c-Kit-positive gastrointestinal stromal tumor (GIST), for which imatinib is also approved for therapy, none of these radioligands were clinically validated.

**Figure 11:**
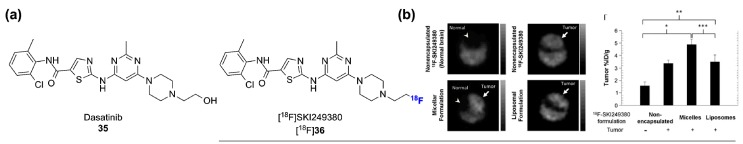
(**a**) Chemical structure of dasatinib and [^18^F]SKI249380 (deoxy-[^18^F]fluoro-dasatinib); (**b**) Axial PET images representing the brain uptake for different nanoformulations of [^18^F]SKI249380 in a PDGFR-driven mouse model of high grade glioma. * *p* = 0.002, ** *p* = 0.014, *** *p* = 0.016. (reprinted from [132], with permission from Elsevier).

Type-I Abl inhibitors such as dasatinib (Sprycel^®^, Bristol-Myers Squibb) have also been approved for clinical applications. Contrary to imatinib, dasatinib is a highly promiscuous inhibitor, especially within the tyrosine kinase class [27,28]. [^18^F]SKI249380, a potent ^18^F-fluorodeoxy dasatinib derivative, was initially validated in K562 tumor xenografts and in a dosimetry study (Figure 11a) [129,130]. Tumor uptakes were similar to [^18^F]SKI696 using the same preclinical paradigm (~1%ID/g). However, [^18^F]SKI249380 is currently under investigation in a clinical trial for potential diagnostic imaging in a wide range of solid tumors [131]. Although the promiscuity of [^18^F]SKI249380, which likely mimics that of dasatinib, may lead to a wider applicability of the tracer, it may likely be challenging in this context to extrapolate reliable data regarding specific kinase biomarkers. However, such an encompassing study design may help to experimentally identify select cases where a multi-targeted probe like [^18^F]SKI249380 can be applicable. The results of this trial will provide a first insight into the clinical potential of radiolabeled promiscuous kinase inhibitors for diagnostic PET imaging.

This ^18^F-dasatinib derivative has also been shown to favorably image CNS tumors in a PDGFB driven model of high-grade glioma using a nanocarrier-encapsulated formulation platform (4.9% ± 0.9%ID/g, 60 min p.i. with micelle encapsulation, and 3.5% ± 0.6%ID/g, 60 min p.i. with liposome encapsulation Figure 11b) [132]. A liposome nanoparticle encapsulation strategy was also demonstrated to be favorable by Medina and colleagues [100] in the imaging of A431 xenografts with the EGFR inhibitor [^124^I]SKI 243. Those results constitute some of the highest tumor uptakes observed in preclinical models so far with radiolabled kinase inhibitors. Hence, liposomal or micellar radiotracer delivery may be a favorable avenue for radiolabled kinase inhibitors imaging [133].

**Figure 12:**
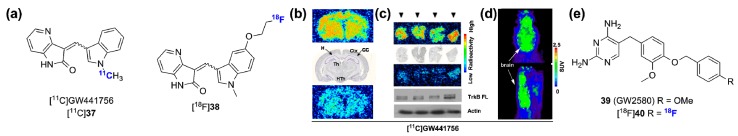
Trk radiolabeled inhibitors. (**a**) Chemical structures of [^11^C]GW441756 and [^18^F]**38**; (**b**,**c**) *In vitro* validation of [^11^C]GW441756 using rat brain and TrkB-expressing human neuroblastoma cryosections (reprinted with permission from [134] © 2015 American Chemical Society); (**d**) PET images of [^11^C]GW441756 in the rat brain; (**e**) Chemical structure of [^18^F]**40**.

**Figure 13:**
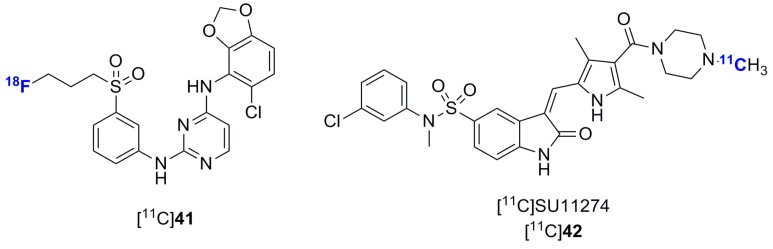
Chemical structures of other radiolabeled tyrosine kinase inhibitors.

Other efforts for the *in vivo* imaging of tyrosine kinases have been directed at the tropomyosin receptor kinases family (TrkA, TrkB and TrkC) (Figure 12) [134,135], the mesenchymal-epithelial transition receptor (MET) [136] and Ephrin type-B receptor 4 (EphB4) (Figure 13) [137].

The TrkA/B/C family consists of three structurally analogous tyrosine kinases with pivotal significance in embryonic development and post-natal maintenance of the mammalian nervous system as well as in neurodegenerative diseases [138]. Those receptors are associated with aggressive tumor phenotypes in a set of neurogenic and non-neurogenic neoplasms including neuroblastoma and pancreatic cancer and constitute an emerging kinase class targeted in clinical trials using kinase inhibitors [139]. Two recent studies have reported radiolabeled inhibitors targeting TrkA/B/C for dual application for CNS Trk expression assessment and potentially tumor imaging. A series of derivatives of the highly potent and selective pan-Trk inhibitor GW441756 were designed and synthesized. Two radiotracers ensuing from a structure activity relationship study were labeled and evaluated (Figure 12a) [134]. Both tracers showed highly specific accumulation in rat brain and TrkB-expressing human neuroblastoma sections *in vitro* (Figure 12b,c). While [^11^C]GW441756 displayed a favorable *in vivo* distribution in healthy rats with good brain penetration (SUV_max_ = 2.0, Figure 12d), [^18^F]**38** was unstable and extensively defluorinated *in vivo*. No reduction in brain accumulation of [^11^C]GW441756 under the tested blocking condition was observed. In a distinct study, a fluorinated derivative of the selective diaminopyrimidine dual CSF-1R/pan-Trk inhibitor GW2580 was identified and radiolabeled ([^18^F]**40**, Figure 12e) [135]. Although no *in vivo* data were provided, [^18^F]**40** was shown to retain the pronounced selectivity of GW2580 [27,28] and constitutes one of the most selective radiolabeled kinase inhibitors identified to date (no additional activity at 3 μM on a 442 kinases panel). Both [^11^C]GW441756 and [^18^F]**40** are lead tracers for Trk PET imaging.

Single tracers for EphB4 and MET have also been reported for tumor imaging. Compound [^18^F]**41** did not show tumor uptake in EphB4-overexpressing tumors *vs.* control despite good metabolic stability. The authors of this study have suggested that the lack of tumor uptake could be due to a lack of selectivity combined with insufficient potency [136]. The second generation MET inhibitor SU11274 was also labeled and evaluated in a MET-positive NCI-H1975 xenograft. The tumor uptakes were only 1.35-fold higher compared to the uptake in a MET-negative NCI-H520 xenograft model and overall very low (<0.4 SUV). This tracer was not further investigated since its original publication [136].

### 2.2. Radiolabeled Serine/Threonine Kinase Inhibitors

**Figure 14:**
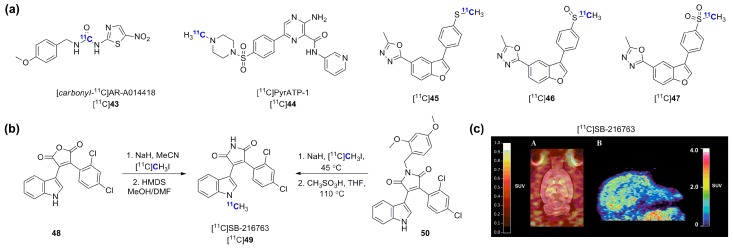
(**a**) Chemical structures of representative novel GSK-3 radiolabeled inhibitors; (**b**) Two radiosynthetic approaches for the synthesis of [^11^C]SB-216763; (**c**) Rodent (**A**) and primate (**B**) brain PET images of [^11^C]SB-216763 (reprinted with permission from [155] © 2015 American Chemical Society).

Serine and threonine kinases include various potential and validated therapeutic targets with key roles in human cancers (e.g., B-Raf [140], PIM kinases [141], aurora kinases [142], mammalian target of rapamycin (mTOR) [143]) and in the pathophysiology of neurodegenerative conditions including Alzheimer’s and Parkinson’s diseases (e.g., glycogen synthase kinase-3 (GSK-3) [144]). Early work towards radiolabeled serine/threonine kinases were centered mostly on poorly selective, staurosporine-based protein kinase A, B and C inhibitors [81,145,146,147,148]. Currently, GSK-3α/β constitute the most pursued serine/threonine targets with different research groups developing radiolabeled inhibitors for CNS imaging. Early work by Vasdev *et al.* led to the synthesis of [*methyl*-^11^C]AR-A014418 [149]. Despite poor brain penetration of [*methyl*-^11^C]AR-A014418, observed both at baseline and in the presence of a P-gp inhibitor [31,149], a second radiosynthesis, used as a platform to establish a novel approach to unsymmetrical [*carbonyl*-^11^C]ureas using [^11^C]CO_2_, was devised and led to the synthesis of [*carbonyl*-^11^C]AR-A014418 (Figure 14a) [150]. This approach relies on the reaction of a reactive amine with [^11^C]CO_2_ followed by dehydration using POCl_3_ and subsequent addition of a second amine component [151].

In a distinct study, Cole *et al.*, reported the synthesis and *in vivo* evaluation of [^11^C]PyrATP-1, a high affinity selective pyrazine-based GSK-3β inhibitor [152]. In spite of apparently favorable properties for brain penetration, [^11^C]PyrATP-1 failed to display any appreciable brain uptake in both rodents and rhesus macaques. In a recent study, Kumata and colleagues have investigated a series a three novel GSK-3 β^11^C-labeled inhibitors corresponding to the different oxidation states of a benzofuran scaffold, specifically [^11^C]methylsulfanyl ([^11^C]**45**), [^11^C]methylsulfunyl ([^11^C]**46**) and [^11^C]methylsulfonyl ([^11^C]**47**) derivatives [153]. This study found a 2-fold higher brain penetration for two out of the three tested tracers in a mouse model of cold water stress associated with increased GSK-3β expression. The study also provided a preliminary validation of the potential of those tracers for imaging ([^11^C]**45** and ([^11^C]**47**). In addition to those studies, two groups have worked towards the synthesis and *in vivo* validation of [^11^C]SB-216763, the radiolabeled version of a tool GSK-3 maleimide inhibitor. The synthesis of [^11^C]SB-216763 was first reported using a two steps approach making use of a maleic anhydride precursor followed by conversion into the corresponding maleimide once the ^11^C-methylation was completed on the indole moiety (Figure 14b) [154]. Although reported RCYs with this approach were 20%–30% (d.c.), no *in vivo* data were provided as part of this study. Then, Li *et al.*, provided a detailed radiosynthetic investigation which led to the development of a more reliable route towards [^11^C]SB-216763 in light of problems reproducing the previous synthetic method due to a [3 + 3]-sigmatropic shift observed when starting from precursor **48** as previously described [155]. Therefore, [^11^C]SB-216763 was obtained from the protected precursor **50**. Although this method only delivers [^11^C]SB-216763 in 1% RCY (185–200 MBq non d.c., 150–350 GBq/μmol), this was sufficient to carry out a detailed preclinical imaging study which revealed that the radiotracer shows good brain penetration in both rodent and primate (SUV_max rodent_ = 2.5, 3 min p.i. and SUV_max rodent_ = 1.9, 5 min p.i., Figure 14c). No blocking data were provided. Nevertheless, [^11^C]SB-216763 is currently the primary lead for GSK-3 PET imaging.

**Figure 15:**
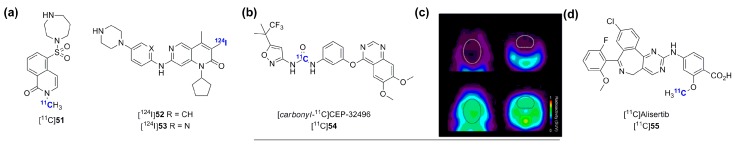
(**a**) Chemical structures of recently identified and evaluated radiolabeked serine/threonine kinase inhibitors; (**b**) Chemical structure of [*carbonyl*-^11^C]CEP-32496; (**c**) Brain PET images of [*carbonyl*-^11^C]CEP-32496 in wild type (top panel) and P-gp/BCRP knockout mice (bottom panel) (reprinted from [160] © 2014, with permission from Elsevier); (**d**) Chemical structure of [^11^C]alisertib.

In recent years, a number of other serine/threonine kinase targets have been investigated using radiolabeled inhibitors and characterized *in vivo*, including rho-kinases (ROCKs), cyclin-dependent kinase 4 (CDK4), B-Raf and most recently aurora kinase A (AURKA). The radiosynthesis of the ROCKs inhibitor [^11^C]**51** (*N*-[^11^C]methyl-hydroxyfasudil), a derivative from the potent Rho-kinases fasudil, was first reported by Valdivia *et al.* (Figure 15a) [156]. This radiotracer was then used in a preclinical *in vivo* model for brain imaging and in a preliminary *in vitro* study measuring Rho kinase activity in hypertrophied cardiomyocytes [157,158]. Although [^11^C]**51** displayed limited applicability for brain imaging due to poor brain penetration [157], it was shown to correlate with Rho-kinase expression *in vitro* in hypertrophied cardiomyocytes and may be appropriate in this context [158]. The ^124^I-labeled Cdk4 inhibitors [^124^I]**52** and [^124^I]**53** were also synthesized and characterized as potential tumor imaging agents due to the role of Cdks in cell proliferation in cancer. Unfortunately, negligible tumor uptake was observed in FaDu tumor xenografts [159]. The clinical validation of other serine/threonine kinases such as B-raf with driver mutation V600E has motivated the development and subsequent PET biodistribution study in mice of [*carbonyl*-^11^C]CEP-32496 ([^11^C]**54**) [160], an isotopologue of an inhibitor currently in clinical development and more recently [^11^C]vemurafenib (Figure 15b) [161]. The inhibitor [*carbonyl*-^11^C]CEP-32496 was synthesized using [^11^C]phosgene and brain accumulation was shown to be significantly influenced by ABCB1/ABCG2 using wild-type and *Abcb1a/1b*;*Abcg2*^−/−^ mice (8-fold higher accumulation in *Abcb1a/1b*;*Abcg2*^−/−^ *vs.* wild-type mice) (Figure 15c) [160]. Most recently, Goos *et al.*, described the first radiolabeled aurora kinase A inhibitor using the selective phase 3 inhibitor alisertib (MLN8237) (Figure 15d) [162]. The radiotracer was characterized in three tumor models (A431, HCT116 and MKN45) and *Abcb1a/1b^−/−^* mice. [^11^C]Alisertib accumulated in xenografts according to AURKA expression levels with highest albeit still modest tumor uptake and tumor-to-background ratios in A431 tumor (1.9% ± 0.2%ID/g at 25 p.i., 2.3 ± 0.8 ratio at 90 min p.i.). In addition, both cellular assays using [^3^H]alisertib and PET imaging in *Abcb1a/1^−/−^* mice with [^11^C]alisertib indicated that alisertib is a P-gp substrate.

**Figure 16:**
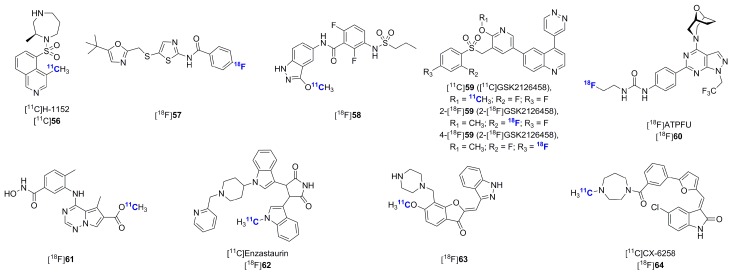
Chemical structures of recently synthesized serine/threonine kinase inhibitors.

Multiple additional serine/threonine kinase inhibitors have been recently radiolabeled. These have been aimed at both previously investigated and novel targets including Rho-kinases, Cdk2, PI3K, mTOR, p38α mitogen-activated protein kinase, PKC and PIM1 (Figure 16) [163,164,165,166,167,168,169,170,171]. However, these reports have only described radiosynthetic work without *in vitro* or *in vivo* validations so far.

## 3. Concluding Remarks

Significant progress and diversification has been achieved in recent years in the field of radiolabeled kinase inhibitors for PET imaging. Recent research has illustrated advances towards prospective imaging applications including: (1) the clinical validation of target expression as a potential predictor of patients’ response to kinase inhibitor treatment using isotopologues of approved inhibitors; (2) the preclinical validation of tumor or tissue engagement during drug development using advanced radiolabeled leads and (3) the visualization and quantification of kinases for tumor, CNS and cardiac imaging with tool inhibitors exclusively developed as radiotracers, outside of a drug development rationale. Despite promising clinical proof-of-concept studies, the successful application for topic (1) remains largely to be established. However, the usefulness of radiolabeled kinase inhibitors for drug development (2) and for the validation of kinase expression and density in preclinical settings (3) has become increasingly evident in recent years.

So far, only isotopologues of approved inhibitors or advanced leads have reached a clinical evaluation stage. While the radiolabeling of approved compounds enjoys the obvious advantages of a facilitated clinical translation, such a strategy presents some shortcomings as illustrated recently. The potentially negative influence of non-optimized or suboptimal ABCB1/ABCG2 efflux, often encountered within approved kinase inhibitors, was illustrated preclinically with the example of the development of [^11^C]erlotinib despite initial promising imaging results. As far as EGFR imaging is concerned, reliable PET data should be attainable with novel generations of inhibitors such as [^11^C]AZD3759. Such compounds will likely be investigated and should enable both peripheral and CNS metastasis imaging, although it may also lead to unwanted brain exposure during peripheral tumor imaging. To avoid problems relating to saturable ABCB1/ABCG2 transporters, the inclusion of imaging experiments using *Abcb1a/1b*;*Abcg2*^−/−^ mice in early developmental steps was shown to be a valuable tool. Selectivity is another parameter which is often suboptimal amongst approved inhibitors when considering PET imaging applications. Although a promiscuous or multi-targeted inhibitor may accumulate in a predefined tumor model, ultimately, tumor heterogeneity compounded with the lack of selectivity is likely to lead to tumor uptake data which will be challenging to interpret. Most kinase inhibitors are approved for patients with tumors overexpressing specific kinases or harboring specific mutations, even though the kinase inhibitor treatment may lead to cross interactions with other oncologically relevant or non-relevant kinases. Ultimately in this context, PET imaging with non-selective inhibitors will potentially limit any useful information that could be gained regarding expression levels specifically related to a biomarker on which the treatment decision should be made. Whereas the development of inhibitors for therapy based on the multi-targeted selectivity principle is established and may offer a safe compromise between efficacy and potential toxicity, this principle is difficult to reconcile with the microdosing nature of PET imaging experiments [172]. Furthermore, such polypharmacology may have unpredictable *in vivo* outcomes at microdose levels due to the wide range of K_M ATP_ values found among kinases. Selective kinase inhibitors have been identified for various kinase targets and could constitute a more favorable starting point in the development of radiolabeled inhibitors [173]. Those compounds exist, but are normally not pursued as therapeutics due to a high risk of inefficacy following mutation events. The wider availability of comprehensive kinase screening should also facilitate compound selection in terms of selectivity for radiotracer development.

The question of whether or not type II inhibitors tend to exhibit better selectivity than type I inhibitors has been subjected to some debate recently [7,10,174]. However, an important observation to bear in mind, as illustrated by the seminal work of Knight and Shokat [26], is that type II inhibitors are less susceptible to ATP competition ensuing from the high intracellular ATP concentration (1–5 mM) compared to type I inhibitors. This is mirrored by K_M ATP_ values, which reflect the affinity for a given kinase towards ATP, and which have been measured for different kinases in both the activated and inactivated states. More generally, K_M ATP_ values among the kinome vary widely. Whereas numerous protein kinases show K_M ATP_ values in the low μM range, other display values in some instances up to a 1000-fold higher (for example mTOR). Those discrepancies are important for the development of ATP competitive radiolabeled inhibitors as they imply that certain kinase targets will be intrinsically more challenging to image than others. Type II inhibitors were also shown to display longer residence times compared to type I inhibitors [175,176,177]. However, it is also important to recognize that type II inhibitors tend to engage the allosteric site via a strong H-bond-based interaction with the DFG motif which often results in additional polar moieties (e.g., ureas, amides). In theory, this can be detrimental for membrane and BBB penetration at microdosing within the timeframe of PET experiments. While non-ATP competitive inhibitors would be advantageous for imaging, such compounds a rare and often do not display sufficient affinity for imaging applications [178,179]. In brief, those challenges relating to binding modes should be explored in more detail in coming years as they may have a strong impact on the outcome and the success of radiolabeled kinase inhibitors.

Finally, it is important to recognize that radiolabeled kinase inhibitors for oncological applications have attained relatively marginal tumor-to-background ratios compared to other radiotracer classes in the same context (e.g., metabolic radiotracers, radiolabeled antibodies and radiolabeled peptides). Further efforts in this area should be encouraged and recent studies illustrating the efficacy of nanoformulations and multivalency derivatization using the longer lived isotope copper-64 should be evaluated carefully and explored. The intrinsic lipophilicity of small molecule kinase inhibitors and high uptakes in excretory organs of such compounds is also likely to limit the application of this class of imaging agents to visualize metastatic lesions, especially in the liver. Importantly, the large chemical space covered by kinase inhibitors is at the same time an enormous opportunity and a possible liability for radiotracer development. The knowledge that only a minuscule fraction of those compounds will be suitable as radiotracers should motivate careful and stringent selection criteria, with the inclusion of considerations well beyond the important questions of radiolabel position, metabolic susceptibility and favorable basic physico-chemical properties. As discussed, these considerations should include the nature of the target in relation with K_M ATP_, the nature of the binding mode selected, the selectivity profile and the efflux transporter susceptibility (such as ABCB1/ABCG2). That being said, those challenges will likely be addressed as part of future advances. The current rapid expansion of kinase inhibitors as a drug class for cancer treatment and the emerging opportunities outside oncology should offer ample areas to further validate the utility of radiolabeled kinase inhibitors in coming years.

## References

[1] Zhang J., Yang P.L., Gray N.S. (2009). Targeting cancer with small molecules kinase inhibitors. Nat. Rev. Cancer.

[2] Capdeville R., Buchdunger E., Zimmermann J., Matter A. (2002). Glivec (STI571, imatinib), a rationally developed, targeted anticancer drug. Nat. Rev. Drug Discov..

[3] Wu P., Nielson T.E., Clausen M.H. (2015). FDA-approved small-molecule kinase inhibitors. Trends Pharmacol. Sci..

[4] U.S. Department of Health and Human Services. U.S. Food and Drug Administration. http://www.fda.gov/NewsEvents/Newsroom/PressAnnouncements/ucm455862.htm.

[5] Van Linden O.P.J., Kooistra A.J., Leurs R., Esch I.J.P., de Graaf C. (2014). KLIFS: A knowledge-based structural database to navigate kinase-ligand interaction space. J. Med. Chem..

[6] Bamborough P., Drewry D., Harper G., Smith G.K., Schneider K. (2008). Assessment of chemical coverage of kinome space and its implications for kinase drug discovery. J. Med. Chem..

[7] Hu Y., Furtmann N., Bajorath J. (2015). Current compound coverage of the kinome. J. Med. Chem..

[8] Cui J.J. (2014). A new challenging and promising era of tyrosine kinase inhibitors. ACS Med. Chem. Lett..

[9] Taylor S.S., Kornev A.P. (2011). Protein kinases: Evolution of dynamic regulatory proteins. Trends Biochem. Sci..

[10] Vijayan R.S.K., He P., Modi V., Duong-Ly K.C., Ma H., Peterson J.R., Dunbrack R.L., Levy R.M. (2015). Conformational analysis of the DFG-out kinase motif and biochemical profiling of structurally validates type II inhibitors. J. Med. Chem..

[11] Barf T., Kaptein A. (2012). Irreversible protein kinase inhibitors: Balancing the benefits and risks. J. Med. Chem..

[12] Liu Q., Sabnis Y., Zhao Z., Zhang T., Buhrlage S.J., Jones L.H., Gray N.S. (2013). Developing irreversible inhibitor of the protein kinase cysteinome. Chem. Biol..

[13] Cox K.J., Shomin C.D., Ghosh I. (2010). Tinkering outside the kinase ATP box: Allosteric (type IV) and bivalent (type V) inhibitors of protein kinases. Future Med. Chem..

[14] Fang Z., Grutter C., Rauh D. (2013). Strategies for the selective regulation of kinases with allosteric modulators: Exploiting structural features. ACS Chem. Biol..

[15] Josephs D.H., Fisher D.S., Spicer J., Flanagan R.J. (2013). Clinical pharmacokinetics of tyrosine kinase inhibitors: Implication for therapeutic drug monitoring. Ther. Drug Monit..

[16] Barouch-Bentov R., Sauer K. (2011). Mechanism of drug resistance in kinases. Expert Opin. Investig. Drugs.

[17] Zhu A., Lee D., Shim H. (2011). Metabolic PET imaging in cancer detection and therapy response. Semin. Oncol..

[18] Matthews P.M., Rabiner E.A., Passchier J., Gunn R.N. (2012). Positron emission tomography molecular imaging for drug development. Br. Clin. Pharmacol..

[19] Cunha L., Szigeti K., Mathe D., Metello L.F. (2014). The role of molecular imaging in modern drug development. Drug Discov. Today.

[20] Benz M.R., Herrmann K., Walter F., Garon E.B., Reckamp K.L., Figlin R., Phelps M.E., Weber W.A., Czernin J., Allen-Auerbach M.S. (2011). (18F)-FDG PET/CT for monitoring treatment responses to the epidermal growth factor receptor inhibitor erlotinib. J. Nucl. Med..

[21] Van Gool M.H., Aukema T.S., Hartemink K.J., Valdes Olmos R.A., van Tinteren H., Klomp H.M. (2014). FDG-PET/CT response evaluation during EGFR-TKI treatment in patients with NSCLC. World J. Radiol..

[22] Caldarella C., Muoio B., Isgro M.A., Porfiri E., Treglia G., Giovanella L. (2014). The role of fluorine-18-fluorodeoxyglucose positron emission tomography in evaluating the response to tyrosine-kinase inhibitors in patients with metastatic primary renal cell carcinoma. Radiol. Oncol..

[23] Sharma R., Aboagye E. (2011). Development of radiotracers for oncology—The interface with pharmacology. Br. J. Pharmacol..

[24] Pike V.W. (2009). PET radiotracers: crossing the blood-brain barrier and surviving metabolism. Trends Pharmacol. Sci..

[25] Slobbe P., Poot A.J., Windhorst A.D., van Dongen G.A.M.S. (2012). PET imaging with small-molecule tyrosine kinase inhibitors: TKI-PET. Drug Discov. Today.

[26] Knight Z.A., Shokat K.M. (2005). Features of selective kinase inhibitors. Chem. Biol..

[27] Davis M.I., Hunt J.P., Herrgard S., Ciceri P., Wodicka L.M., Pallares G., Hocker M., Treiber D.K., Zarrinkar P.P. (2011). Comprehensive analysis of kinase inhibitor selectivity. Nat. Biotechnol..

[28] Anastassiadis T., Deacon S.D., Devarajan K., Ma H., Peterson J.R. (2011). Comprehensive assay of kinase catalytic activity reveals features of kinase inhibitor selectivity. Nat. Biotechnol..

[29] Zhang L., Villalobos A., Beck E.M., Bocan T., Chappie T.A., Chen L., Grimwood S., Heck S.D., Helal C.J., Hou X. (2013). Design and selection parameters to accelerate the discovery of novel central nervous system positron emission tomography (PET) ligands and their application in the development of a novel phosphodiesterase 2A PET ligand. J. Med. Chem..

[30] Van de Bittner G.C., Ricq E.L., Hooker J.M. (2014). A philosophy for CNS radiotracer design. Acc. Chem. Res..

[31] Hicks J.W., VanBrocklin H.F., Wilson A.A., Houle S., Vasdev N. (2010). Radiolabeled small molecule protein kinase inhibitors for imaging with PET or SPECT. Molecules.

[32] Mulholland G.K., Zheng Q.H., Winkle W.L., Carlson K.A. (1997). Synthesis and biodistribution of new C-11 and F-18 labeled epidermal growth factor receptor ligands. J. Nucl. Med..

[33] DeJesus O.T., Murali D., Flores L.G., Converse A.K., Dick D.W., Oakes T.R., Roberts A.D., Nickles R.J. (2003). Synthesis of [^18^F]-ZD1839 as a PET imaging agent for epidermal growth factor receptors. J. Label. Compd. Radiopharm..

[34] Johnstrom P., Fredriksson A., Thorell J.O., Stone-Elander S. (1998). Synthesis of [methoxy-^11^C]PD153035, a selective EGF receptor tyrosine kinase inhibitor. J. Label. Compd. Radiopharm..

[35] Fredriksson A., Johnstrom P., Thorell J.O., von Heijne G., Hassan M., Eksborg S., Kogner P., Borgstrom P., Ingvar M., Stone-Elander S. (1999). *In vivo* evaluation of the biodistribution of ^11^C-labeled PD153035 in rats without and with neuroblastoma implants. Life Sci..

[36] Samén E., Thorell J.-O., Fredriksson A., Stone-Elander S. (2006). The tyrosine kinase inhibitor PD153035: Implication of labeling position on radiometabolites formed *in vitro*. Nucl. Med. Biol..

[37] Ackermann U., Tochon-Danguy H.J., Scott A.M. (2004). [^11^C]AG1478—A potential PET tracer for the molecular imaging of epidermal growth factor receptor (EGFR). J. Nucl. Med..

[38] Owino N.O., Shao X., Snyder S.E. (2005). Preparation of a reversible EGFR inhibitor, [^11^C]CIRC, as a PET radiotracer for tumor imaging. J. Label. Compd. Radiopharm..

[39] Snyder S.E., Sherman P.S., Blair J.B. (2000). 4-(3-chloro-4-[^18^F]fluorophenylamino)-6,7-dimethoxyquinazoline: A radiolabeled EGF receptor inhibitor for imaging tumor biochemistry with PET. J. Nucl. Med..

[40] Bonasera T.A., Ortu G., Rozen Y., Krais R., Freedman N.M.T., Chisin R., Gazit A., Levitzki A., Mishani E. (2001). Potential ^18^F-labeled biomarkers for epidermal growth factor receptor tyrosine kinase. Nucl. Med. Biol..

[41] Dorff P.N., Vasdev N., O’Neil J.P., Nandanan E., Gibbs A.R., VanBrocklin H.F. (2003). Synthesis of 2′-, 3′-, and 4′-[^18^F]fluoroanilinoquinazoline. J. Label. Compd. Radiopharm..

[42] Ben-David I., Rozen Y., Ortu G., Mishani E. (2003). Radiosynthesis of ML03, a novel positron emission tomography biomarker for targeting epidermal growth factor receptor via the labeling synthon: [^11^C]acryloyl chloride. Appl. Radiat. Isot..

[43] Vasdev N., Dorff P.N., Gibbs A.R., Nandanan E., Reid L.M., O’Neill J.P., VanBrocklin H.F. (2005). Synthesis of 6-acrylamido-4-(2-[^18^F]fluoroanilino)quinazoline: A prospective irreversible EGFR binding probe. J. Label. Compd. Radiopharm..

[44] Vasdev N., Dorff P.N., Gibbs A.R., Nandanan E., Reid L.M., O’Neil J.P., VanBrocklin H.F. (2004). Synthesis of 6-acrylamido-4-(2-[^18^F]fluoro-anilino)quinazoline. J. Nucl. Med..

[45] Waldherr C., Satyamurthy N., Toyokuni T., Wang S., Mellinghoff I., Tran C., Stout D., Halpern B., Silverman D.H., Barrio J.R., Phelps M.E., Sawyers C.L., Czernin J. (2003). Evaluation of *N*-{4-[(3′-[^18^F]fluoroethylphenyl)amino]-6-quinazolinyl} acrylamide ([^18^F]FEQA), a labeled tyrosine kinase inhibitor, for imaging epidermal growth factor receptor density. J. Nucl. Med..

[46] Abourbeh G., Dissoki S., Jacobson O., Litchi A., Ben Daniel R., Laki D., Levitzki A., Mishani E. (2007). Evaluation of radiolabeled ML04, a putative irreversible inhibitor of epidermal growth factor receptor, as a bioprobe for PET imaging of EGFR-overexpressing tumors. Nucl. Med. Biol..

[47] Mishani E., Abourbeh G., Rozen Y. (2004). Novel carbon-11 labeled 4-dimethylamino-but-2-enoic acid [4-(phenylamino)-quinazoline-6-yl]-amides: Potential PET bioprobes for molecular imaging of EGFR-positive tumors. Nucl. Med. Biol..

[48] Dissoki S., Eshet R., Billauer H., Mishani E. (2009). Modified PEG-anilinoquinazoline derivatives as potential EGFR PET agents. J. Label. Compd. Radiopharm..

[49] Pantaleo M., Mishani E., Nanni C., Landuzzi L., Boschi S., Nicoletti G., Dissoki S., Paterini P., Piccaluga P., Lodi F. (2010). Evaluation of modified Evaluation of modified PEG-anilinoquinazoline derivatives as potential agents for EGFR imaging in cancer by small animal PET. Mol. Imaging Biol..

[50] Dissoki S., Aviv Y., Laky D., Abourbeh G., Levitzki A., Mishani E. (2007). The effect of the [^18^F]- PEG group on tracer qualification of [4-(phenylamino)-quinazoline-6-yl]-amide moiety—An EGFR putative irreversible inhibitor. Appl. Radiat. Isot..

[51] Kobus D., Giesen Y., Ullrich R., Backes H., Neumaier B. (2009). A fully automated two-step synthesis of an 18F-labelled tyrosine kinase inhibitor for EGFR kinase activity imaging in tumors. Appl. Radiat. Isot..

[52] VanBrocklin H.F., Vasdev N., Dorff P.N., O’Neil J.P., Taylor S.E. (2003). Metabolism of [^18^F]fluoroanilinoquinazolines by human hepatocytes. J. Label. Compd. Radiopharm..

[53] Chong C.R., Janne P.A. (2013). The quest to overcome resistance to EGFR-targeted therapies in cancer. Nat. Med..

[54] Gazdar A.F. (2009). Activating and resistance mutations of EGFR in non-small-cell lung cancer: Role in clinical response to EGFR tyrosine kinase inhibitors. Oncogene.

[55] Seimbille Y., Phelps M.E., Czernin J., Silverman D.H.S. (2005). Fluoriue-18 labeling of 6,7-disubstituted anilinoquinazoline derivatives for positron emission tomography (PET) imaging of tyrosine kinase receptors: Synthesis of F-18-Iressa and related molecular probes. J. Label. Comp. Radiopharm..

[56] Su H., Seimbille Y., Ferl G.Z., Bodenstein C., Fueger B., Kim K.J., Hsu Y.T., Dubinett S.M., Phelps M.E., Czernin J. (2008). Evaluation of F-18 Gefitinib as a molecular imaging probe for the assessment of the epidermal growth factor receptor status in malignant tumors. Eur. J. Nucl. Mol. Imaging.

[57] Wang J.Q., Gao M.Z., Miller K.D., Sledge G.W., Zheng Q.H. (2006). Synthesis of [C-11]Iressa as a new potential PET cancer imaging agent for epidermal growth factor receptor tyrosine kinase. Bioorg. Med. Chem..

[58] Holt D.P., Ravert H.T., Dannals R.F., Pomper M.G. (2006). Synthesis of [C-11]Gefitinib for imaging epidermal growth factor receptor tyrosine kinase with positron emission tomography. J. Label. Compd. Radiopharm..

[59] Zhang M.R., Kumata K., Hatori A., Takai N., Toyohara J., Yamasaki T., Yanamoto K., Yui J., Kawamura K., Koike S., Ando K., Suzuki K. (2010). [^11^C]Gefitinib ([^11^C]Iressa): Radiosynthesis, *in vitro* uptake, and *in vivo* imaging of intact murine fibrosarcoma. Mol. Imaging Biol..

[60] Lappchen T., Vlaming M.L., Custers E., Lub J., Sio C.F., DeGroot J., Steinbach O.C. (2012). Automated synthesis of [^18^F]Gefitinib on a modular system. Appl. Radiat. Isot..

[61] Elkind N.B., Szentpetery Z., Apari A., Ozvegy-Laczka C., Varady G., Ujhelly O., Szabó K., Homolya L., Váradi A., Buday L. (2005). Multidrug transporter ABCG2 prevents tumor cell death induced by the epidermal growth factor receptor inhibitor Iressa (ZD1839, gefitinib). Cancer Res..

[62] Zeng Q., Wang J., Cheng Z., Chen K., Johnstrom P., Varnas K., Li D.Y., Yang Z.F., Zhang X. (2015). Discovery and evaluation of clinical candidate AZD3759, a potent, oral active, central nervous system-penetrant, epidermal growth factor receptor tyrosine kinase inhibitor (EGFR TKI). J. Med. Chem..

[63] Kawamura K., Yamasaki T., Yui J., Hatori A., Konno F., Kumata K., Irie T., Fukumura T., Suzuki K., Kanno I. (2009). In vivo evaluation of p-glycoprotein and breast cancer resistance protein modulation in the brain using [^11^C]Gefitinib. Nucl. Med. Biol..

[64] Vlaming M.L.H., Lappchen T., Jansen H.T., Kivits S., van Driel A., van de Steeg E., van der Hoorn J.W., Sio C.F., Steinbach O.C., DeGroot J. (2015). PET-ET imaging with [^18^F]-gefitinib to measure Abcb1a/1b (P-gp) and Abcg2 (Bcrp1) mediated drug-drug interaction at the murine blood-brain barrier. Nucl. Med. Biol..

[65] ClinicalTrials.gov. https://clinicaltrials.gov/ct2/show/NCT02228369.

[66] Dowell J., Minna J.D., Kirkpatrick P. (2005). Erlotinib hydrochloride. Nat. Rev. Drug Discov..

[67] Memon A.A., Jakobsen S., Dagnaes-Hansen F., Sorensen B.S., Keiding S., Nexo E. (2009). Positron emission tomography (PET) imaging with C-11 -labeled Erlotinib: A micro-PET study on mice with lung tumor xenografts. Cancer Res..

[68] Petrulli J.R., Sullivan J.M., Zheng M.Q., Bennett D.C., Charest J., Huang Y., Morris E.D., Contessa J.N. (2013). Quantitative analysis of [^11^C]-Erlotinib PET demonstrates specific binding for activating mutations of the EGFR kinase domain. Neoplasia.

[69] Abourbeh G., Itamar B., Salnikov O., Beltsov S., Mishani E. (2015). Identifying Erlotinib-sensitive non-small cell lung carcinoma tumors in mice using [^11^C]Erlotinib PET. EJNMMI Res..

[70] Memon A.A., Weber B., Winterdahl M., Jakobsen S., Meldgaard P., Madsen H.H.T., Keiding S., Nexo E., Sorensen B.S. (2011). PET imaging of patients with non-small cell lung cancer employing an egf receptor targeting drug as tracer. Br. J. Cancer.

[71] Bahce I., Smit E.F., Lubberink M., van der Veldt A.A.M., Yaqub M., Windhorst A.D., Schuit R.C., Thunnissen E., Heideman D.A.M., Postmus P.E. (2013). Development of [^11^C]erlotinib positron emission tomography for *in vivo* evaluation of EGF receptor mutation status. Clin. Cancr Res..

[72] Weber B., Winterdahl M., Memon A., Sorensen B.S., Keiding S., Sorensen L., Nexo E., Meldgaard P. (2011). Erlotinib accumulation in brain metastases from non-small cell lung cancer: Visualization by positron emission tomography in a patient harboring a mutation in the epidermal growth factor receptor. J. Thorac. Oncol..

[73] Traxl A., Wanek T., Mairinger S., Stanek J., Filip T., Sauberer M., Muller M., Kuntner C., Langer O. (2015). Breast cancer resistance protein and p-glycoprotein influence *in vivo* disposition of ^11^C-erlotinib. J. Nucl. Med..

[74] Slobbe P., Windhorst A.D., Stigter-van Walsum M., Smit E.F., Niessen H.G., Solca F., Stehle G., van Dongen G.A., Poot A.J. (2015). A comparative PET imaging study with the reversible and irreversible EGFR tyrosine kinase inhibitors [^11^C]Erlotinib and [^18^F]Afatinib in lung cancer-bearing mice. EJNMMI Res..

[75] Li D., Ambrogio L., Shimamura T., Kubo S., Takahashi M., Chirieac L.R., Padera R.F., Shapiro G.I., Baum A., Himmelsbach F. (2008). BIBW2992, an irreversible EGFR/HER2 inhibitor highly effective in preclinical lung cancer models. Oncogene.

[76] Slobbe P., Windhorst A.D., Stigter-van Walsum M., Schuit R.C., Smit E.F., Niessen H.G., Solca F., Stehle G., van Dongen G.A., Poot A.J. (2014). Development of [^18^F]afatinib as new TKI-PET tracer for EGFR positive tumors. Nucl. Med. Biol..

[77] European Medicines Agency (2013). Committee for Medicinal Products for Human Use (CHMP) Assessment Report for Giotrif (afatinib). http://www.ema.europa.eu/docs/en_GB/document_library/EPAR_-_Product_Information/human/002280/WC500152392.pdf.

[78] Copeland R.A. (2010). The dynamics of drug-target interactions: Drug-target residence time and its impact on efficacy and safety. Expert Opin. Drug Discov..

[79] Johnson D.S., Weerapana E., Cravatt B.F. (2010). Strategies for discovering and derisking covalent, irreversible enzyme inhibitors. Future Med. Chem..

[80] Bos M., Mendelsohn J., Kim Y.M., Albanell J., Fry D.W., Baselga J. (1997). PD153035, a tyrosine kinase inhibitor, prevents epidermal growth factor receptor activation and inhibits growth of cancer cells in a receptor number-dependent manner. Clin. Cancer Res..

[81] Sasaki T., Ishii S.I., Senda M., Akinaga S., Murakata C. (1996). Synthesis of [7β−methoxy ^11^C]methoxy staurosporine for imaging protein kinase C localization in the brain. Appl. Radiat. Isot..

[82] Wang H., Yu J.M., Yang G., Song X., Sun X., Zhao S., Mu D. (2007). Assessment of ^11^C-labeled-4-*N*-(3-bromoanilino)-6,7-dimethoxyquinazoline as a positron emission tomography agent to monitor epidermal growth factor receptor expression. Cancer Sci..

[83] Wang H., Yu J.M., Yang G.R., Song X.R., Sun X.R., Zhao S.Q., Wang X.W., Zhao W. (2007). Further characterization of the epidermal growth factor receptor ligand ^11^C-PD153035. Chin. Med. J..

[84] Meng X., Yu J.M., Yang G.R., Zhao S.Q., Sun X.D.l. (2008). ^11^C-PD153035 PET/CT for molecular imaging of EGFR in patients with non-small cell lung cancer (NSCLC) [abstract]. J. Clin. Oncol..

[85] Liu N., Li M., Li X., Meng X., Yang G., Zhao S., Yang Y., Ma L., Fu Z., Yu J. (2009). PET-based biodistribution and radiation dosimetry of epidermal growth factor receptor-selective tracer ^11^C-PD153035 in humans. J. Nucl. Med..

[86] Meng X., Loo B.W., Ma L., Murphy J.D., Sun X., Yu J. (2011). Molecular imaging with ^11^C-PD153035 PET/CT predicts survival in non-small cell lung cancer treated with EGFR-TKI: A pilot study. J. Nucl. Med..

[87] Samen E., Arnberg F., Lu L., Olofsson M.H., Tegnebratt T., Thorell J.O., Holmin S., Stone-Elander S. (2013). Metabolism of epidermal growth factor receptor targeting probe [^11^C]PD153035: Impact on biodistribution and tumor uptake in rats. J. Nucl. Med..

[88] Sun J., Cai L., Zhang K., Zhang A., Pu P., Yang W., Gao S. (2014). A pilot study on EGFR-targeted molecular imaging of PET/CT with tracer ^11^C-PD153035 in human gliomas. Clin. Nucl. Med..

[89] Basuli F., Wu H., Li C., Shi Z.-D., Sulima A., Griffiths G.L. (2011). A first synthesis of ^18^F-radiolabeled Lapatinib: A potential tracer for positron emission tomographic imaging of erbb1/erbb2 tyrosine kinase activity. J. Label. Compd. Radiopharm..

[90] Saleem A., Searle G.E., Kenny L.M., Huiban M., Kozlowski K., Waldman A.D., Woodley L., Palmieri C., Lowdell C., Kaneko T. (2015). Lapatinib access into normal brain and brain metastases in patients with HER-2 overexpressing breast cancer. EJNMMI Res..

[91] Polli J.W., Humphreys J.E., Harmon K.A., Castellino S., O’Mara M.J., Olson K.L., John-Williams L.S., Koch K.M., Serabjit-Singh C.J. (2008). The role of efflux and uptake transporters in [*N*-{3-chloro-4-[(3-fluorobenzyl)oxy]phenyl}-6-[5-({[2-(methylsulfonyl)ethyl]amino}methyl)-2-furyl]-4-quinazolinamine (GW572016, lapatinib) disposition and drug interactions. Drug Metab. Dispos..

[92] Pivot X., Manikhas A., Z’urawski B., Chmielowska E., Karaszewska B., Allerton R., Chan S., Fabi A., Bidoli P., Gori S. (2015). CEREBEL (EGF111438): A Phase III, Randomized, Open-Label Study of Lapatinib Plus Capecitabine Versus Trastuzumab Plus Capecitabine in Patients With Human Epidermal Growth Factor Receptor 2–Positive Metastatic Breast Cancer. J. Clin. Oncol..

[93] Pisaneschi F., Nguyen Q.-D., Shamsaei E., Glaser M., Robins E., Kaliszczak M., Smith G., Spivey A.C., Aboagye E.O. (2010). Development of a new epidermal growth factor receptor positron emission tomography imaging agent based on the 3-cyanoquinoline core: Synthesis and biological evaluation. Bioorg. Med. Chem..

[94] Pisaneschi F., Slade R.L., Iddon L., George G.P., Nguyen Q.D., Spivey A.C., Aboagye E.O. (2014). Synthesis of a new fluorine-18 glycosylated “click” cyanoquinoline for the imaging of epidermal growth factor receptor. J. Label. Comp. Radiopharm..

[95] Pal A., Balatoni J., Mukhopadhyay U., Ogawa K., Gonzalez-Lepera C., Shavrin A., Volgin A., Tong W., Alauddin M., Gelovani J. (2011). Radiosynthesis and initial *in vitro* evaluation of [^18^F]FPEG6-IPQA—A novel PET radiotracer for imaging EGFR expression-activity in lung carcinomas. Mol. Imaging Biol..

[96] Yeh H.H., Ogawa K., Balatoni J., Mukhapadhyay U., Pal A., Gonzalez-Lepera C., Shavrin A., Soghomonyan S., Flores L., Young D. (2011). Molecular imaging of active mutant l858R EGF receptor (EGFR) kinase-expressing nonsmall cell lung carcinomas using PET/ct. Proc. Natl. Acad. Sci. USA.

[97] Yeh S.H., Lin C.F., Kong F.L., Wang H.E., Hsieh Y.J., Gelovani J.G., Liu R.S. (2013). Molecular imaging of nonsmall cell lung carcinomas expressing active mutant EGFR kinase using PET with [^124^I]-morpholino-ipqa. Biomed. Res. Int..

[98] Wang M., Gao M., Zheng Q.H. (2014). The first radiosynthesis of [^11^C]AZD8931 as a new potential PET agent for imaging of EGFR, HER2 and HER3 signaling. Bioorg. Med. Chem. Lett..

[99] Vasdev N., Dorff P.N., O’Neil J.P., Chin F.T., Hanrahan S., VanBrocklin H.F. (2011). Metabolic stability of 6,7-dialkoxy-4-(2-, 3- and 4-[^18^F]fluoroanilino)quinazolines, potential EGFR imaging probes. Bioorg. Med. Chem..

[100] Medina O.P., Pillarsetty N., Glekas A., Punzalan B., Longo V., Gonen M., Zanzonico P., Smith-Jones P., Larson S.M. (2011). Optimizing tumor targeting of the lipophilic EGFR-binding radiotracer SKI 243 using a liposomal nanoparticle delivery system. J. Control. Release.

[101] Neto C., Fernandes C., Oliveira M.C., Gano L., Mendes F., Kniess T., Santos I. (2012). Radiohalogenated 4-anilinoquinazoline-based EGFR-TK inhibitors as potential cancer imaging agents. Nucl. Med. Biol..

[102] Chang S., Zhang L., Xu S., Luo J., Lu X., Zhang Z., Xu T., Liu Y., Tu Z., Xu Y. (2012). Design, synthesis, and biological evaluation of novel conformationally constrained inhibitors targeting epidermal growth factor receptor threonine⁷⁹⁰→methionine⁷⁹⁰ mutant. J. Med. Chem..

[103] Han C., Huang Z., Zheng C., Wan L., Zhang L., Peng S., Ding K., Ji H., Tian J., Zhang Y. (2013). Novel hybrids of (phenylsulfonyl)furoxan and anilinopyrimidine as potent and selective epidermal growth factor receptor inhibitors for intervention of non-small-cell lung cancer. J. Med. Chem..

[104] Heald R.A., Chan B.K., Bryan C., Eigenbrot C., Yu C., Burdick D., Hanan E.J., Chan E., Schaefer G., La H. (2015). Noncovalent Mutant Selective Epidermal Growth Factor Receptor Inhibitors: A Lead Optimization Case History. J. Med. Chem..

[105] Zhou W., Ercan D., Chen L., Yun C.-H., Li D., Capelletti M., Cortot A.B., Chirieac L., Iacob R.E., Padera R. (2009). Novel mutant-selective EGFR kinase inhibitors against EGFR T790M. Nature.

[106] Kumar R., Crouthamel M.-C., Rominger D.H., Gontarek R.R., Tummino1 P.J., Levin R.A., King A.G. (2009). Myelosuppression and kinase selectivity of multikinase angiogenesis inhibitors. Br. J. Cancer.

[107] Hu-Lowe D.D., Zou H.Y., Grazzini M.L., Hallin M.E., Wickman G.R., Amundson K., Chen J.H., Rewolinski D.A., Yamazaki S., Wu E.Y. (2008). Nonclinical Antiangiogenesis and Antitumor Activities of Axitinib (AG-013736), an Oral, Potent, and Selective Inhibitor of Vascular Endothelial Growth Factor Receptor Tyrosine Kinases 1, 2, 3. Clin. Cancer Res..

[108] Boss D.S., Glen H., Beijnen J.H., Keesen M., Morrison R., Tait B., Copalu W., Mazur A., Wanders J., O’Brien J.P., Schellens J.H.M., Evans T.R.J. (2012). A phase I study of E7080, a multitargeted tyrosine kinase inhibitor, in patients with advanced solid tumours. Br. J. Cancer.

[109] Herbst R.S., Heymach J.V., O’Reilly M.S., Onn A., Ryan A.J. (2007). Vandetanib (ZD6474): An orally available receptor tyrosine kinase inhibitor that selectively targets pathways critical for tumor growth and angiogenesis. Expert Opin. Investig. Drugs.

[110] Peters J.-U. (2013). Polypharmacology—Foe or friend. J. Med. Chem..

[111] Li F., Jiang S., Zu Y., Lee D.Y., Li Z. (2014). A tyrosine kinase inhibitor-based high-affinity PET radiopharmaceutical targets vascular endothelial growth factor receptor. J. Nucl. Med..

[112] Samen E., Lu L., Mulder J., Thorell J.O., Damberg P., Tegnebratt T., Holmgren L., Rundqvist H., Stone-Elander S. (2014). Visualization of angiogenesis during cancer development in the polyoma middle t breast cancer model: Molecular imaging with (R)-[^11^C]PAQ. EJNMMI Res..

[113] Gao M.Z., Lola C.M., Wang M., Miller K.D., Sledge G.W., Zheng Q.H. (2011). Radiosynthesis of C-11 Vandetanib and C-11 chloro-Vandetanib as new potential PET agents for imaging of VEGFR in cancer. Bioorg. Med. Chem. Lett..

[114] Asakawa C., Ogawa M., Kumata K., Fujinaga M., Kato K., Yamasaki T., Yui J., Kawamura K., Hatori A., Fukumura T. (2011). [^11^C]Sorafenib: Radiosynthesis and preliminary PET study of brain uptake in p-gp/bcrp knockout mice. Bioorg. Med. Chem. Lett..

[115] Poot A.J., van der Wildt B., Stigter-van Walsum M., Rongen M., Schuit R.C., Hendrikse N.H., Eriksson J., van Dongen G.A., Windhorst A.D. (2013). [^11^C]Sorafenib: Radiosynthesis and preclinical evaluation in tumor-bearing mice of a new tki-PET tracer. Nucl. Med. Biol..

[116] Caballero J., Muñoz C., Alzate-Morales J.H., Cunha S., Gano L., Bergmann R., Steinbach J., Kniess T. (2012). Synthesis, in silico, *in vitro*, and *in vivo* investigation of 5-[¹¹C]methoxy-substituted sunitinib, a tyrosine kinase inhibitor of VEGFR-2. Eur. J. Med. Chem..

[117] Slobbe P., Windhorst A.D., Haumann R., Schuit R., van Dongen G.A., Poot A.J. (2015). Development of an LC-MS method to analyze the primary metabolite of [^11^C]nintedanib *in vivo*. J. Label. Compd. Radiopharm..

[118] Ilovich O., Billauer H., Dotan S., Mishani E. (2010). Labeled 3-aryl-4-indolylmaleimide derivatives and their potential as angiogenic PET biomarkers. Bioorg. Med. Chem..

[119] Ilovich O., Åberg O., Långström B., Mishani E. (2009). Rhodium-mediated [^11^C]carbonylation: A library of *n*-phenyl-*n*′-{4-(4-quinolyloxy)-phenyl}-[^11^c]-urea derivatives as potential pet angiogenic probes. J. Label. Compd. Radiopharm..

[120] Dissoki S., Abourbeh G., Salnikov O., Mishani E., Jacobson O. (2015). PET molecular imaging of angiogenesis with a multiple tyrosine kinase receptor-targeted agent in a rat model of myocardial infarction. Mol. Imaging Biol..

[121] Fernandes C., Santos I.C., Santos I., Pietzsch H.-J., Kunstler J.-U., Kraus W., Rey A., Margaritis N., Bourkoula A., Chiotellis A. (2008). Rhenium and technetium complexes bearing quinazoline derivatives: Progress towards a ^99m^Tc biomarker for EGFR-TK imaging. Dalton Trans..

[122] Jørgensen J.T., Persson M., Madsen J., Kjær A. (2013). High tumor uptake of ^64^Cu: implications for molecular imaging of tumor characteristics with copper-based PET tracers. Nucl. Med. Biol..

[123] Lagas J.S., van Waterschoot R.A., Sparidans R.W., Wagenaar E., Beijnen J.H., Schinkel A.H. (2010). Breast cancer resistance protein and *P*-glycoprotein limit sorafenib brain accumulation. Mol. Cancer Ther..

[124] Kil K.-E., Ding Y.-S., Lin K.-S., Alexoff D., Kim S.W., Shea C., Xu Y., Muench L., Fowler J.S. (2007). Synthesis and positron emission tomography studies of carbon-11-labeled Imatinib (Gleevec). Nucl. Med. Biol..

[125] Peng Z., Bornman W., Pal A., Ghosh P., Lim S.T., Gelovani J., Maxwell D., Alauddin M.M. STI571 analogs: ^18^F-STI571as potential agents for PET imaging of c-kit expression at a kinase level. Proceedings of the 233rd ACS National Meeting.

[126] Doubrovin M., Kochetkova T., Santos E., Veach D.R., Smith-Jones P., Pillarsetty N., Balatoni J., Bornmann W., Gelovani J., Larson S.M. (2010). (124)i-iodopyridopyrimidinone for PET of abl kinase-expressing tumors *in vivo*. J. Nucl. Med..

[127] Glekas A.P., Pillarsetty N.K., Punzalan B., Khan N., Smith-Jones P., Larson S.M. (2011). *In vivo* imaging of bcr-abl overexpressing tumors with a radiolabeled Imatinib analog as an imaging surrogate for Imatinib. J. Nucl. Med..

[128] Peng Z., Maxwell D.S., Sun D., Bhanu Prasad B.A., Pal A., Wang S., Balatoni J., Ghosh P., Lim S.T., Volgin A. (2014). Imatinib analogs as potential agents for PET imaging of bcr-abl and c-kit expression at a kinase level. Bioorg. Med. Chem..

[129] Veach D.R., Namavari M., Pillarsetty N., Santos E.B., Beresten-Kochetkov T., Lambek C., Punzalan B.J., Antczak C., Smith-Jones P.M., Djaballah H. (2007). Synthesis and biological evaluation of a fluorine-18 derivative of Dasatinib. J. Med. Chem..

[130] Dunphy M.P., Zanzonico P., Veach D., Somwar R., Pillarsetty N., Lewis J., Larson S. (2012). Dosimetry of 18F-labeled tyrosine kinase inhibitor ski-249380, a Dasatinib-tracer for PET imaging. Mol. Imaging Biol..

[131] ClinicalTrials.gov. https://clinicaltrials.gov/ct2/show/NCT01916135.

[132] Benezra M., Hambardzumyan D., Penate-Medina O., Veach D.R., Pillarsetty N., Smith-Jones P., Phillips E., Ozawa T., Zanzonico P.B., Longo V. (2012). Fluorine-labeled Dasatinib nanoformulations as targeted molecular imaging probes in a pdgfb-driven murine glioblastoma model. Neoplasia.

[133] Chakravarty R., Hong H., Cai W. (2014). Positron emission tomography image-guided drug delivery: Current status and future perspectives. Mol. Pharm..

[134] Bernard-Gauthier V., Aliaga A., Aliaga A., Boudjemeline M., Hopewell R., Kostikov A., Rosa-Neto P., Thiel A., Schirrmacher R. (2015). Syntheses and evaluation of carbon-11 and fluorine-18-radiolabeled pan-tropomyosin receptor kinase (Trk) inhibitors : Exploration of the 4-aza-2-oxindole scaffold as Trk PET imaging agents. ACS Chem. Neurosci..

[135] Bernard-Gauthier V., Schirrmacher R. (2014). 5-(4-((4-[^18^F]fluorobenzyl)oxy)-3-methoxybenzyl)pyrimidine-2,4-diamine: A selective dual inhibitor for potential PET imaging of Trk/CSF-1R. Bioorg. Med. Chem. Lett..

[136] Wu C., Tang Z., Fan W., Zhu W., Wang C., Somoza E., Owino N., Li R., Ma P.C., Wang Y. (2010). *In vivo* positron emission tomography (PET) imaging of mesenchymal-epithelial transition (MET) receptor. J. Med. Chem..

[137] Mamat C., Mosch B., Neuber C., Köckerling M., Bergmann R., Pietzsch J. (2012). Fluorine-18 radiolabeling and radiopharmacological characterization of a benzodioxolylpyrimidine-based radiotracer targeting the receptor tyrosine kinase EphB4. ChemMedChem.

[138] Huang E.J., Reichardt L.F. (2003). Trk receptors: Roles in neuronal signal transduction. Annu. Rev. Neurosci..

[139] Vaishnavi A., Le A.T., Doebele R.C. (2015). TRKing down an old oncogene in a new era of targeted therapy. Cancer Discov..

[140] Dienstmann R., Tabernero J. (2011). BRAF as a target for cancer therapy. Anticancer Agents Med. Chem..

[141] Blanco-Aparicio C., Carnero A. (2013). Pim kinases in cancer: Diagnostic, prognostic and treatment opportunities. Biochem. Pharmacol..

[142] Gavriilidis P., Giakoustidis A., Giakoustidis D. (2015). Aurora Kinases and Potential Medical Applications of Aurora Kinase Inhibitors: A Review. J. Clin. Med. Res..

[143] Ciuffreda L., di Sanza C., Incani U.C., Milella M. (2010). The mTOR pathway: A new target in cancer therapy. Curr. Cancer Drug Targets.

[144] Arfeen M., Bharatam P.V. (2013). Design of glycogen synthase kinase-3 inhibitors: An overview on recent advancements. Curr. Pharm. Des..

[145] Vasdev N., LaRonde F.J., Woodgett J.R., Garcia A., Rubie E.A., Meyer J.H., Houle S., Wilson A.A. (2008). Rationally designed PKA inhibitors for positron emission tomography: Synthesis and cerebral biodistribution of *N*-(2-(4-bromocinnamylamino)ethyl)-*N*-[^11^C]methyl-isoquinoline-5-sulfonamide. Bioorg. Med. Chem..

[146] Wang M., Gao M., Miller K.D., Sledge G.W., Hutchins G.D., Zheng Q.-H. (2010). The first design and synthesis of [^11^C]MKC-1 ([^11^C]Ro 31–7453), a new potential PET cancer imaging agent. Nucl. Med. Biol..

[147] Takahashi K., Kudo K., Okada M., Yanamoto K., Hatori A., Irie T., Suzuki K., Miura S. (2007). Synthesis and biodistribution of [^11^C]methyl-bisindolylmareimide III, an inhibitor of protein kinase C. J. Label. Compd. Radiopharm..

[148] Cai L., Ozaki H., Fujita M., Hong J.S., Bukhari M., Innis R.B., Pike V.W. (2009). [^11^C]GO6976 as a potential radioligand for imaging protein kinase C with PET. J. Label. Compd. Radiopharm..

[149] Vasdev N., Garcia A., Stableford W.T., Young A.B., Meyer J.H., Houle S., Wilson A.A. (2005). Synthesis and *ex vivo* evaluation of carbon-11 labelled *N*-(4-methoxybenzyl)-]*N*′-(5-nitro-1,3-thiazol-2-yl)urea ([C-11]AR-A014418): A radiolabelled glycogen synthase kinase-3 beta specific inhibitor for PET studies. Bioorg. Med. Chem..

[150] Hicks J.W., Wilson A.A., Rubie E.A., Woodgett J.R., Houle S., Vasdev N. (2012). Towards the preparation of radiolabeled 1-aryl-3-benzyl ureas: Radiosynthesis of [^11^C-carbonyl] AR-A014418 by [^11^C]CO_2_ fixation. Bioorg. Med. Chem. Lett..

[151] Rotstein B.H., Liang S.H., Holland J.P., Collier T.L., Hooker J.M., Wilson A.A., Vasdev N. (2013). ^11^CO2 fixation: A renaissance in PET radiochemistry. Chem. Commun..

[152] Cole E.L., Shao X., Sherman P., Quesada C., Fawaz M.V., Desmond T.J., Scott P.J. (2014). Synthesis and evaluation of [^11^C]pyrATP-1, a novel radiotracer for PET imaging of glycogen synthase kinase-3beta (GSK-3β). Nucl. Med. Biol..

[153] Kumata K., Yui J., Xie L., Zhang Y., Nengaki N., Fujinaga M., Yamasaki T., Shimoda Y., Zhang M.R. (2015). Radiosynthesis and preliminary PET evaluation of glycogen synthase kinase 3beta (GSK-3β) inhibitors containing [^11^C]methylsulfanyl, [^11^C]methylsulfinyl or [^11^C]methylsulfonyl groups. Bioorg. Med. Chem. Lett..

[154] Wang M., Gao M., Miller K.D., Sledge G.W., Hutchins G.D., Zheng Q.H. (2011). The first synthesis of [^11^C]SB-216763, a new potential PET agent for imaging of glycogen synthase kinase-3 (GSK-3). Bioorg. Med. Chem. Lett..

[155] Li L., Shao X., Cole E.L., Ohnmacht S.A., Ferrari V., Hong Y.T., Williamson D.J., Fryer T.D., Quesada C.A., Sherman P., Riss P.J. (2015). Synthesis and initial *in vivo* studies with [^11^C]SB-216763: The first radiolabeled brain penetrative inhibitor of GSK-3. ACS Med. Chem. Lett..

[156] Valdivia A.C., Mason S., Collins J., Buckley K.R., Coletta P., Beanlands R.S., Dasilva J.N. (2010). Radiosynthesis of *N*-[^11^C]-methyl-hydroxyfasudil as a new potential PET radiotracer for rho-kinases (ROCKs). Appl. Radiat. Isot..

[157] Taniguchi J., Seki C., Takuwa H., Kawaguchi H., Ikoma Y., Fujinaga M., Kanno I., Zhang M.R., Kuwabara S., Ito H. (2014). Evaluation of rho-kinase activity in mice brain using *N*-[^11^C]methyl-hydroxyfasudil with positron emission tomography. Mol. Imaging Biol..

[158] Moreau S., DaSilva J.N., Valdivia A., Fernando P. (2015). *N*-[^11^C]-Methyl-hydroxyfasudil is a potential biomarker of cardiac hypertrophy. Nucl. Med. Biol..

[159] Koehler L., Graf F., Bergmann R., Steinbach J., Pietzsch J., Wuest F. (2010). Radiosynthesis and radiopharmacological evaluation of cyclin-dependent kinase 4 (CDK4) inhibitors. Eur. J. Med. Chem..

[160] Shimoda Y., Yui J., Fujinaga M., Xie L., Kumata K., Ogawa M., Yamasaki T., Hatori A., Kawamura K., Zhang M.R. (2014). [^11^C-carbonyl]CEP-32496: Radiosynthesis, biodistribution and PET study of brain uptake in p-gp/bcrp knockout mice. Bioorg. Med. Chem. Lett..

[161] Slobbe P., Windhorst A.D., van Dongen G.A., Poot A.J. (2015). Synthesis of [^11^C]vemurafenib via a unique [^11^C]CO carbonylative Stille coupling to image V600E mutated B-Raf in cancer. J. Label. Compd. Radiopharm..

[162] Goos J.A., Verbeek J., Geldof A.A., Hiemstra A.C., van de Wiel M.A., Adamzek K.A., Delis-Van Diemen P.M., Stroud S.G., Bradley D.P., Meijer G.A. (2015). Molecular imaging of aurora kinase A (AURKA) expression: Synthesis and preclinical evaluation of radiolabeled alisertib (MLN8237). Nucl. Med. Biol..

[163] Suzuki M., Takashima-Hirano M., Koyama H., Yamaoka T., Sumi K., Nagata H., Hidaka H., Doi H. (2012). Efficient synthesis of [^11^C]H-1152, a PET probe specific for Rho-kinases, highly potential targets in diagnostic medicine and drug development. Tetrahedron.

[164] Svensson F., Kniess T., Gergmann R., Pietzsch J., Wuest F. (2011). Synthesis of an 18F-labeled cyclin-dependant kinase-2 inhibitor. J. Label. Compd. Radiopharm..

[165] Wang M., Gao M., Miller K.D., Zheng Q.H. (2013). Synthesis of 2,6-difluoro-*N*-(3-[^11^C]methoxy-1*H*-pyrazolo[3,4-*b*]pyridine-5-yl)-3-(propylsulfonamidio)benzamide as a new potential PET agent for imaging of B-Raf^V6^°°^E^ in cancers. Bioorg. Med. Chem. Lett..

[166] Wang M., Gao M., Miller K.D., Sledge G.W., Zheng Q.H. (2012). [^11^C]GSK2126458 and [^18^F]GSK2126458, the first radiosynthesis of new potential PET agents for imaging of PI3K and mTOR in cancers. Bioorg. Med. Chem. Lett..

[167] Majo V.J., Simpson N.R., Prabhakaran J., Mann J.J., Kumar J.S. (2014). Radiosynthesis of [^18^F]ATPFU: A potential PET ligand for mTOR. J. Label. Comp. Radiopharm..

[168] Wang M., Gao M., Zheng Q.H. (2014). Synthesis of carbon-11-labeled 4-(phenylamino)-pyrrolo[2,1-f][1,2,4]triazine derivatives as new potential PET tracers for imaging of p38α mitogen-activated protein kinase. Bioorg. Med. Chem. Lett..

[169] Wang M., Xu L., Gao M., Miller K.D., Sledge G.W., Zheng Q.H. (2011). [^11^C]enzastaurin, the first design and radiosynthesis of a new potential PET agent for imaging of protein kinase C. Bioorg. Med. Chem. Lett..

[170] Gao M., Wang M., Miller K.D., Zheng Q.H. (2013). Synthesis of (*Z*)-2-((1h-indazol-3-yl)methylene)-6-[^11^C]methoxy-7-(piperazin-1-ylmethyl)benzofuran-3(2H)-one as a new potential PET probe for imaging of the enzyme PIM1. Bioorg. Med. Chem. Lett..

[171] Wang M., Tzintzun R., Gao M., Xu Z., Zheng Q.H. (2015). Synthesis of [^11^C]CX-6258 as a new PET tracer for imaging of pim kinases in cancer. Bioorg. Med. Chem. Lett..

[172] Morph R. (2010). Selectively Nonselective Kinase Inhibition: Striking the Right Balance. J. Med. Chem..

[173] Uitdehaag J.C., Verkaar F., Alwan H., de Man J., Buijsman R.C., Zaman G.J. (2012). A guide to picking the most selective kinase inhibitor tool compounds for pharmacological validation of drug targets. Br. J. Pharmacol..

[174] Zhao Z., Wu H., Wang L., Liu Y., Knapp S., Liu Q., Gray N.S. (2014). Exploration of type II binding mode: A privileged approach for kinase inhibitor focused drug discovery?. ACS Chem. Biol..

[175] Stachel S.J., Sanders J.M., Henze D.A., Rudd M.T., Su H.P., Li Y., Nanda K.K., Egbertson M.S., Manley P.J., Jones K.L. (2014). Maximizing diversity from a kinase screen: Identification of novel and selective pan-Trk inhibitors for chronic pain. J. Med. Chem..

[176] Regan J., Pargellis C.A., Cirillo P.F., Gilmore T., Hickey E.R., Peet G.W., Proto A., Swinamer A., Moss N. (2003). The Kinetics of Binding to p38 MAP Kinase by Analogues of BIRB 796. Bioorg. Med. Chem. Lett..

[177] Pargellis C., Tong L., Churchill L., Cirillo P.F., Gilmore T., Graham A.G., Grob P.M., Hickey E.R., Moss N., Pav S. (2002). Inhibition of p38 MAP Kinase by Utilizing a Novel Allosteric Binding Site. Nat. Struct. Biol..

[178] Gavrin L.K., Saiah E. (2013). Approaches to discover non-ATP site kinase inhibitors. Med. Chem. Commun..

[179] Breen M.E., Soellner M.B. (2014). Small molecule substrate phosphorylation site inhibitors of protein kinases: Approaches and challenges. ACS Chem. Biol..

